# Beyond Beauty: Microalgae and Cyanobacteria as Next-Generation Platforms for Functional Dermocosmetics, Cutaneous Delivery, and Skin Health

**DOI:** 10.3390/md24100359

**Published:** 2026-10-15

**Authors:** Clément Daviaud, Herminia Dominguez, Luana Granja Fernández, María Lourdes Mourelle, Kévin Baranger, Nicolas Bridiau, Laurent Picot

**Affiliations:** 1 UMR CNRS 7266 Littoral Environnement et Sociétés LIENSs, Institut du Littoral et de l’Environnement, La Rochelle Université, 2 Rue Olympe de Gouges, 17000 La Rochelle, France; clement.daviaud@univ-lr.fr (C.D.); kevin.baranger@univ-lr.fr (K.B.); nicolas.bridiau@univ-lr.fr (N.B.); 2 CINBIO, Faculty of Sciences, Campus Ourense, Universidade de Vigo, 32004 Ourense, Spain; herminia@uvigo.gal (H.D.); luana.granja@uvigo.gal (L.G.F.); 3 Vigo Campus Lagoas Marcosende, Universidade de Vigo, 36310 Vigo, Spain; lmourelle@uvigo.es

**Keywords:** microalgae, cyanobacteria, dermocosmetics, cutaneous delivery, exopolysaccharides, mycosporine-like amino acids, extracellular vesicles, photoprotection, skin microbiome, photosynthetic biomaterials

## Abstract

Microalgae and cyanobacteria are valuable sources of bioactive ingredients and innovative biological systems for skin applications. Their metabolites include exopolysaccharides (EPS), mycosporine-like amino acids (MAAs), carotenoids, phycobiliproteins, peptides and lipopeptides, polyunsaturated lipids, chlorophyll derivatives, and distinctive secondary metabolites such as scytonemin. Cyanobacteria additionally provide defined molecular candidates, including sacran, C-phycocyanin, MAAs, scytonemin-related compounds, and specialized peptide scaffolds. These compounds may support hydration and barrier function, photoprotection, control of oxidative stress and inflammation, pigmentation, antimicrobial and antibiofilm activity, extracellular-matrix preservation, and wound repair. This review examines major bioactive families, their sources and mechanisms, with particular emphasis on the strength of evidence supporting skin applications. Recent developments from 2024 to 2026 are highlighted, including microalgal extracellular vesicles (nanoalgosomes), photoactivated antimicrobial systems, photosynthetic hydrogels and microneedle patches, green extraction and biorefinery strategies, reconstructed human skin and skin-on-chip models, and artificial-intelligence-assisted formulation. Most reported effects remain supported mainly by biochemical, cellular, or preclinical evidence, whereas human validation is still limited. Translation will, therefore, depend on rigorous chemical standardization, effective cutaneous delivery, demonstrated stability and safety, scalable manufacturing, and validation in human-relevant models. Microalgae and cyanobacteria thus extend beyond conventional natural extracts toward next-generation dermocosmetic and cutaneous-delivery platforms.

## 1. Introduction: From Algal Ingredients to Functional Cutaneous Platforms

Dermocosmetics is a scientific and commercial term for products intended to support skin appearance and physiology; it is not a separate legal product category in the European Union. Skin combines physical and chemical barrier functions with immune surveillance, microbial colonization, and neuroendocrine signaling. These interacting functions provide a broader rationale for topical product development than appearance alone [1,2,3].

An active ingredient is useful only if it remains stable, is released in the appropriate form, and reaches its intended site at a tolerable exposure. Surface hydration, epidermal photoprotection, and dermal matrix modulation impose different delivery requirements. Classification as a cosmetic or medicinal product depends on intended use, claims, composition, and mode of action rather than on a simple threshold of penetration depth or pharmacological intensity [3,4,5,6].

Microalgae and cyanobacteria are particularly well suited to this approach because adaptation to environmental stress has driven the production of a remarkably diverse range of bioactive metabolites. These include high-molecular-weight extracellular polysaccharides, ultraviolet-absorbing MAAs, carotenoids, chlorophyll derivatives, phycobiliproteins, antimicrobial and signaling peptides, polyunsaturated fatty acids, and unusual secondary metabolites. Two newer modalities expand this repertoire further: extracellular vesicles that behave as naturally assembled nanocarriers, and intact photosynthetic cells that can generate oxygen locally when incorporated into biomaterials. Recent reviews describe this molecular diversity, while primary studies establish the distinct proof-of-concept status of vesicular and living modalities [4,5,7,8,9,10,11].

A major weakness of the field is the frequent jump from biological activity to cosmetic efficacy. Antioxidant, anti-inflammatory, or antimicrobial effects are often demonstrated in cell-free assays, tumor cell lines, macrophages, or other non-cutaneous systems. These results are useful for screening, but they do not establish a skin benefit. Translation requires a relevant cutaneous model, a realistic topical concentration, formulation compatibility, and an endpoint that can support the intended claim. The same distinction is essential for living photosynthetic systems, whose biomedical potential is high but whose safety and regulatory requirements differ fundamentally from those of a conventional cosmetic. The predominance of in vitro evidence in the field and the limited topical human data make this distinction essential [7,12,13].

This review adopts a source-to-function-to-delivery framework. It connects photosynthetic biomanufacturing with the chemistry of major algal bioactive families, maps these functions onto cutaneous targets, evaluates delivery technologies from conventional topical vehicles to nanoalgosomes and living photosynthetic scaffolds, and finally considers the evidence and regulatory requirements needed for credible translation.

### 1.1. Scope, Novelty and Evidence-Grading Approach

This review examines which microalgal and cyanobacterial functions can realistically be delivered to human skin. We distinguish three development stages: opportunities closest to cosmetic application; candidates requiring further validation in human-relevant models and biomedical proof-of-concept technologies whose intended use or mode of action is likely to place them outside standard cosmetic regulation. The distinction reflects the uneven evidence base. A 2023 systematic review identified 151 eligible studies on cosmetic-related microalgal and cyanobacterial bioactivities; 96% used in vitro approaches and only 4% included in vivo work [7].

We use an operational evidence hierarchy to describe proximity to human application. Level A denotes controlled human intervention studies with formulation-specific endpoints, including small exploratory controlled trials; sample size, route of administration, and limitations are stated separately. Level B comprises uncontrolled human studies, including exploratory tolerability studies, ex vivo human skin, and well-controlled reconstructed human skin.

Level C denotes animal studies and non-human organotypic models addressing a skin-relevant mechanism. Level D denotes cellular and biochemical experiments, including monolayers of human skin cells. A plausible mechanism without intervention data remains an ungraded mechanistic concept. These categories are not a validated risk-of-bias score: efficacy, delivery, and tolerability are graded separately, and oral evidence does not establish topical efficacy.

This critical narrative review uses the existing reference corpus as a starting point, supplemented by targeted searches of PubMed/MEDLINE and publisher websites, backward reference checking, and DOI metadata verification through Crossref. The revision searches were updated on 2 October 2026.

Recent work from 2020 onward was emphasized, while earlier structural, cultivation, formulation, and human studies were retained when foundational. Search concepts combined microalgae or cyanobacteria with skin, dermocosmetics, photoprotection, inflammation, microbiome, wound healing, extracellular vesicles, exopolysaccharides, mycosporine-like amino acids, pigments, peptides, topical delivery, photobioreactor, productivity, extraction recovery, and techno-economic analysis.

Peer-reviewed studies were selected when the source organism or material, experimental model, and claim-relevant endpoint could be identified. Primary research supports specific mechanistic and quantitative comparisons; reviews provide context and identify earlier work, and official sources support regulatory statements.

Process comparisons retain their original units, operating conditions, and study boundaries; modeled costs are distinguished from measured performance. Literature selection was purposive rather than exhaustive, without prospective registration, duplicate independent screening, or formal risk-of-bias assessment. Accordingly, this is a critical narrative review, not a systematic review or meta-analysis, and the tables summarize representative examples rather than pooled effects.

### 1.2. Skin as an Interconnected Biological Target

Contemporary cutaneous biology makes simple single-endpoint claims increasingly inadequate. Barrier lipids and tight junctions influence microbial ecology; microbial metabolites shape keratinocyte and immune signaling; sensory neurons respond to cytokines and barrier damage and repeated ultraviolet, pollution or inflammatory stress promotes mitochondrial dysfunction and cellular senescence. A product designed to improve long-term skin resilience, therefore, needs to be evaluated as an intervention in a coupled epithelial-immune-microbial-neural system rather than solely as a source of antioxidant equivalents [1,2,14,15].

This systems perspective is useful because one strain can generate several fractions with different roles. A secreted EPS may remain on the surface and support hydration or reduce adhesion; a lipophilic carotenoid can partition into membranes; a peptide may act on a microbial pathway and extracellular vesicles can carry multiple components into cells. Such complementarity may justify multi-fraction formulations, but only if the fractions are chemically standardized and tested together to determine whether their effects are additive, synergistic, or antagonistic.

Table 1 summarizes the principal microalgal and cyanobacterial bioactive classes discussed in this review, their representative sources, skin-relevant functions, and supporting references.

## 2. From Programmable Cultivation to Downstream Processing: Defining the Active Material

### 2.1. Biodiversity, Stress Physiology and Chemical Opportunity

Microalgae comprise phylogenetically diverse eukaryotic photosynthetic microorganisms, while cyanobacteria are oxygenic photosynthetic prokaryotes. Their taxonomic distance is matched by striking biochemical diversity. Diatoms, chlorophytes, eustigmatophytes, haptophytes, red microalgae and cyanobacteria differ in membrane lipids, light-harvesting systems, cell walls, storage compounds and secondary metabolism. Consequently, the term “algal extract” is chemically uninformative unless species, strain, culture conditions, harvesting stage and extraction process are specified.

Environmental adaptation provides a rational discovery strategy. High-light and ultraviolet stress enrich photoprotective metabolites such as carotenoids, MAAs and, in selected cyanobacteria, scytonemin. Salinity and desiccation can stimulate extracellular polymeric substances that retain water and protect cell surfaces. Nutrient limitation can remodel lipid composition and induce storage products, while interspecies competition can select antimicrobial or quorum-interfering metabolites. These stress responses can be intentionally exploited through controlled cultivation, turning environmental physiology into a form of metabolic elicitation.

### 2.2. Cultivation Strategies and Discovery of Defined Actives

Cultivation must be optimized against recoverable product rather than biomass alone. Growth-associated products such as photosynthetic proteins can benefit from nutrient-replete, semi-continuous operation; light supply, dilution rate, self-shading, and nitrogen availability must support both growth and the desired cellular product fraction. Stress-induced accumulation, exemplified by astaxanthin in *Haematococcus pluvialis*, often benefits from a biomass-growth stage followed by an independently optimized induction stage. This is a physiological distinction rather than an absolute primary-versus-secondary-metabolite rule: some compounds show mixed growth-associated and stress-responsive behavior [57,58,59].

Discovery is most informative when cultivation is linked directly to a target product profile. The OSMAC principle can reveal stress-responsive metabolic states by varying light, nutrients, salinity, temperature, or culture mode, while LC-HRMS metabolomics, molecular networking, transcriptomics, and genome mining connect those states to defined chemical features [60,61,62,63,64].

A barrier-support EPS should be characterized by secretion yield, molecular-mass distribution, charge density, rheology, and preservative compatibility; an MAA platform by spectral coverage, photostability, and composition; carotenoids by isomer distribution and oxidation stability and antimicrobial peptides by potency together with host-cell and commensal selectivity. Synthetic biology can further improve reproducibility through pathway engineering, but engineered and non-engineered routes should ultimately be judged by the same criteria: identity, purity, potency, safety, scalability, and reproducibility [62].

For a steady culture, volumetric product output can be expressed as dilution rate × biomass concentration × product mass fraction, before downstream losses. For a two-stage batch process, productivity must include the full growth-and-induction cycle and the reactor capacity allocated to both stages. A higher pigment percentage is, therefore, not necessarily a higher process productivity. Reactor geometry, optical path, light spectrum and photon flux, gas transfer, temperature, culture volume, and cycle duration should accompany reported yields [57,58,59].

Table 2 contrasts a growth-supporting cyanobacterial process with stress-induced carotenoid production. These laboratory benchmarks illustrate process selection; they are not a scale-independent ranking of reactor performance. For product discovery, a suitable operating window must also preserve the molecular fingerprint, biological potency, and batch reproducibility required for a topical ingredient.

### 2.3. Biorefinery and Downstream Processing: Recovery Without Losing Function

A sustainable algal ingredient should be evaluated across the complete downstream chain rather than on biological origin alone. Drying, cell disruption, solvent consumption, oxygen exposure, and purification can dominate both environmental burden and loss of biological function. A biorefinery can fractionate biomass sequentially so that pigments or lipids, proteins or peptides, extracellular polysaccharides, and residual biomass enter distinct value streams. Secreted products are especially attractive because they may be recovered from culture supernatants without destroying the producing cells.

Pressure-assisted, pulsed-electric-field, ultrasound, microwave, pressurized-liquid, supercritical-fluid, and hybrid approaches can shorten processing time, enable wet-biomass extraction, and reduce solvent use, but each process must be matched to the molecular target [8,60,65]. Pigments require protection from oxygen, light, metals, and heat; phycobiliproteins and peptides are vulnerable to denaturation or proteolysis and aggressive purification can remove cofactors responsible for extract-level activity. Process intensification is, therefore, useful only when recovery yield, energy input, solvent recycling, chemical integrity, and retained biological potency are evaluated together. Table 3 provides quantitative examples for a lipophilic pigment and a water-soluble chromoprotein, which require different separation strategies.

A useful process comparison reports, at each step, the input and output target mass, recovery, concentration or purity, solvent and water consumption, energy use, and retained potency. Membrane rejection is not equivalent to overall process recovery, and an absorbance purity ratio is not a mass-purity percentage. For example, an *Arthrospira maxima* ultrafiltration/diafiltration study reported phycocyanin membrane rejection above 96%, but its final absorbance purity ratio of 1.16 does not mean 116% purity [66].

The preferred downstream route is, therefore, product-dependent. Wet extraction can avoid drying for a lipophilic ingredient if solvent recovery, residues, and oxidation are controlled; aqueous membrane processing can preserve a chromoprotein but requires management of fouling, dilution water, and microbial quality. For EPS, the corresponding decision concerns retention of molecular-mass distribution and charge rather than maximal small-molecule purity. A chemically cleaner fraction is not necessarily more biologically active [19,66,67].

As an explicitly illustrative mass balance, four sequential operations each recovering 90% of the target retain 65.6% overall (0.9 × 0.9 × 0.9 × 0.9); four operations at 95% retain 81.5%. These calculated values are not experimental results. They show why stage-specific recovery and potency losses must be propagated before choosing an extraction technology or assigning a production cost.

**Table 3:** Downstream and economic benchmarks. Measured separation results and modeled facility costs are deliberately separated; the original study units and cost basis are retained.

Material/Evidence Type	Step or Comparison	Quantitative Result	Decision and Boundary
Astaxanthin; measured laboratory recovery [67]	Wet centrifugal partition extraction; 244-mL column; disrupted *H. pluvialis* biomass	85% recovery; 3.5 g oleoresin from 7.85 g biomass	Avoids prior drying; recovery included a cleaning fraction. Oleoresin is not pure astaxanthin.
Astaxanthin; modeled facility [67]	8910 kg biomass/year; in-house CO2 extraction versus wet extraction of disrupted cysts or germinated zoospores	Annual total product costs: EUR 3.81, 2.98, and 2.92 million, respectively; downstream electricity: 290.7, 135.9, and 130.7 MWh/year	2021 study assumptions; hypothetical scale-up, not observed commercial costs or 2026 prices. Upstream and finished-product costs must not be conflated.
Phycocyanin; measured separation [66]	*A. maxima* aqueous extract; 20-kDa membrane; 2.0 ± 0.1 bar; 24 ± 2 °C; six diafiltration cycles	Mean flux 14.4 L m^−2^ h^−1^; membrane rejection >96%; concentration 0.232 to 1.174 mg/mL; purity ratio 0.74 to 1.16; DNA removal 91.7%	Concentration and purity are different metrics; rejection is not whole-process recovery. Water use, fouling and biological potency remain relevant.

### 2.4. Quality-by-Design, Safety and Potency-Linked Standardization

Natural origin does not simplify safety assessment. Cyanobacterial cultures require strain-level identification and exclusion of toxin-producing contaminants; marine biomass may accumulate metals, salts, iodine, or environmental contaminants and extracts can contain residual solvents or inflammatory impurities. The safety object is, therefore, the final ingredient produced by a defined process, not the organism name alone. For extracellular vesicles or living materials, critical quality attributes expand to particle/cargo characterization, viability or containment, storage stability, light-response control, and defined failure modes.

Chemical fingerprinting should be paired with a potency assay whenever an ingredient is intended to be reference grade. An EPS batch, for example, can be released against molecular-mass/charge specifications plus an adhesion or barrier assay; an MAA fraction against LC-UV/MS composition and photoprotection; a pigment against chromatographic integrity and photoactivity and an EV preparation against particle/cargo metrics plus a keratinocyte-response assay. Linking composition to function is more informative than standardization based only on total solids, total phenolics, or species identity.

**Figure 1:**
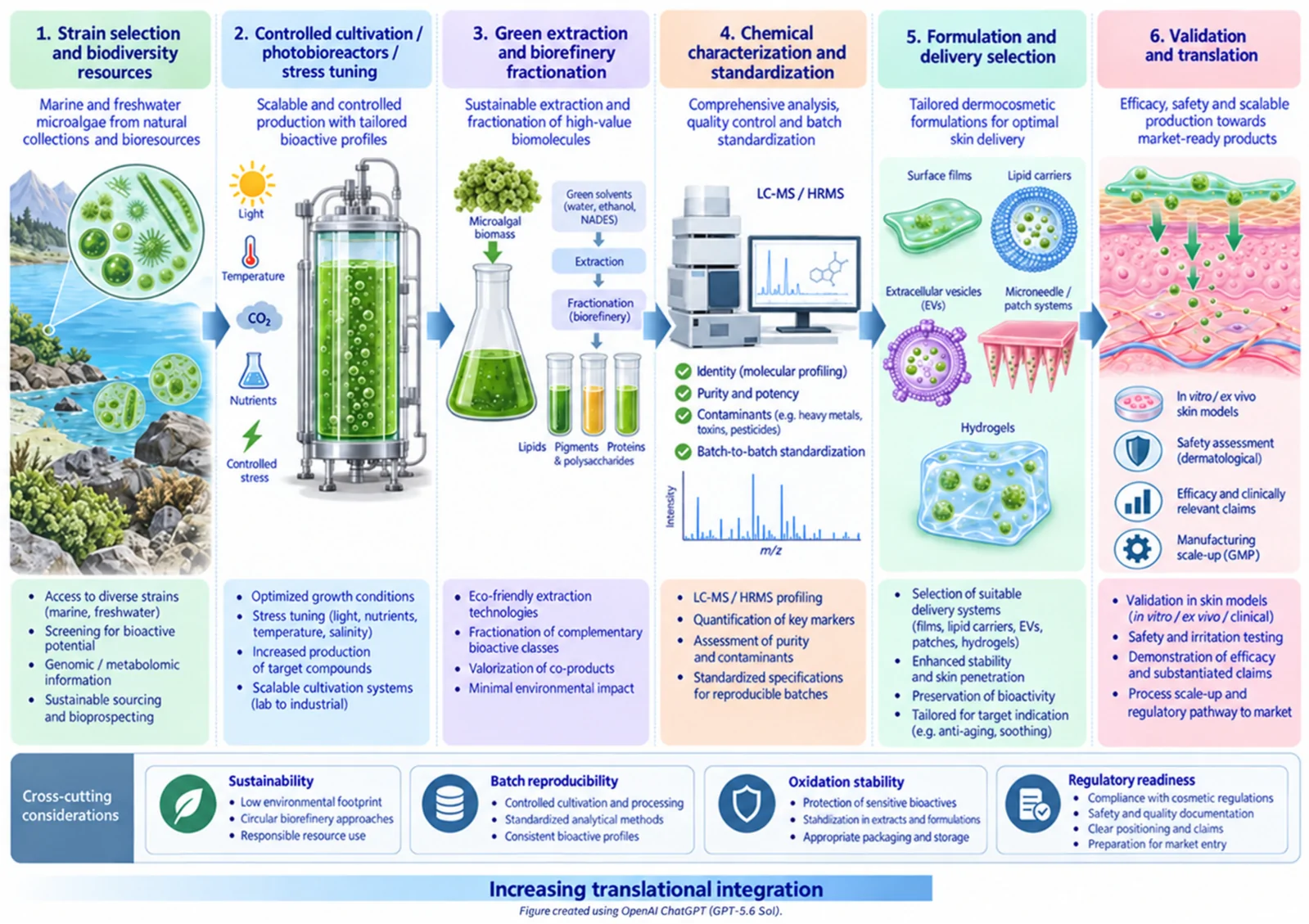
From photosynthetic resource to validated dermocosmetic product. The development pathway progresses from strain selection and controlled cultivation to green extraction/biorefinery, chemical characterization and standardization, formulation and delivery selection, and translational validation. Cross-cutting considerations include sustainability, batch reproducibility, oxidation stability, and regulatory readiness. Figure created using OpenAI ChatGPT (GPT-5.6 Sol).

The overall pathway from photosynthetic resource to validated dermocosmetic product is summarized in Figure 1.

## 3. Molecular Diversity and Structure–Function Relationships

### 3.1. Exopolysaccharides: Hydration, Barrier Support and Biofilm Control

Microalgae and cyanobacteria release extracellular polymeric substances in which polysaccharides are usually dominant. These exopolysaccharides (EPS) can remain soluble in the culture medium or associated with the cell surface and biofilm. They are typically high-molecular-weight heteropolymers containing glucose, galactose, xylose, rhamnose, fucose, mannose and uronic acids in strain-specific proportions, frequently decorated by sulfate or other charged groups. Their high hydration capacity, viscosity and interfacial behavior explain why they can simultaneously function as active ingredients and formulation excipients [68,69,70,71].

EPS function depends on architecture rather than total carbohydrate content alone. Relevant descriptors include linkage sequence, molecular-mass distribution, branching, uronic-acid and sulfate substitution, and supramolecular conformation [68,69,70,71,72,73,74]. In a structurally resolved Nostoc EPS, the backbone is [→4)-β-D-Xylp-(1→4)-β-D-Glcp-(1→4)-α-L-Arap-(1→3)-β-D-Manp-(1→]*n*. Approximately 60% of glucose residues carry C6-linked uronic-acid substitutions, including lactylated derivatives [75].

By contrast, sacran from *Aphanothece sacrum* is a very-high-molecular-weight, sulfated anionic heteropolysaccharide whose heterogeneous composition should not be represented as a uniquely resolved short repeat [76]. These examples distinguish a sequence-resolved polymer segment from compositional and conformational descriptors; neither establishes a universal skin-microbiome-selective motif.

EPS can also provide functions beyond moisturization, but the structure-activity relationships are still incompletely resolved. High anionic charge and dense hydration layers can modify electrostatic and hydrophobic interactions at a surface, while chain length, branching, and adsorbed conformation influence steric repulsion and film persistence. These physicochemical effects provide a plausible basis for anti-adhesion without requiring bactericidal activity.

Consistent with this interpretation, EPS fractions from *Cylindrotheca closterium* and *Tetraselmis suecica* changed surface hydrophilicity and reduced bacterial adhesion in a strain- and fraction-dependent manner [20]. The data do not yet support a universal motif that confers microbiome selectivity; rather, they show that extraction-dependent composition and interfacial properties can change adhesion behavior. This distinction is important for skin applications, where ecological modulation may be preferable to broad killing.

Secreted EPS may also offer a processing advantage: in some systems, they can be recovered without destroying the producing cells. Continuous or semi-continuous harvesting could, therefore, avoid cell disruption and provide a high-value biorefinery stream, although concentration and water-removal costs remain. Cultivation stress can modify EPS yield and properties, potentially enabling production of polymers with predefined characteristics [70,73]. Cultivation, recovery, and downstream processing all contribute to final EPS quality [74].

### 3.2. Mycosporine-like Amino Acids and Photoprotection

MAAs are small, water-soluble secondary metabolites characterized by a cyclohexenone or cyclohexenimine chromophore conjugated to amino-acid or amino-alcohol substituents. Their absorption maxima span approximately 268–362 nm, allowing different members of the family to cover complementary portions of the UV spectrum. Shinorine, porphyra-334, mycosporine-glycine, palythine and related compounds are produced by diverse marine and terrestrial phototrophs and have evolved primarily as photoprotective molecules [77,78,79].

The photophysical behavior of MAAs is unusually attractive for topical photoprotection. They absorb ultraviolet energy efficiently and dissipate most of it non-radiatively as heat with little photosensitized ROS generation. In addition, cell-based studies associate selected MAAs with direct antioxidant activity, modulation of inflammatory signaling, reduced matrix-degrading responses and protection against UV-induced cellular damage. Mycosporine-glycine-containing preparations have also been linked to wound-repair responses in skin models [80,81].

MAAs should not be reduced to the label “natural UV filters.” Purified shinorine, porphyra-334, and mycosporine-glycine from *Sphaerospermopsis torques-reginae* were well tolerated by human dermal fibroblasts at the tested concentrations and showed strong UVB-protective activity; in that specific cell assay, shinorine outperformed benzophenone [24]. A 2025 patent review also shows growing interest in MAA-containing skincare formulations [82]. These findings support MAA-based photoprotective ingredients, but they do not establish sunscreen efficacy in humans. At the same time, broader bioprotective applications and synthetic MAA- or gadusol-inspired analogs are expanding the design space beyond direct extraction from biomass [83,84].

Structural diversification may further broaden topical performance. A glycosylated MAA, 13-O-(β-galactosyl)-porphyra-334, isolated from *Nostoc sphaericum*, protected human keratinocytes against UVA- and UVB-associated damage and showed radical-scavenging activity, illustrating how glycosylation can expand the MAA structure-function space [29].

Claims of “DNA repair” require particular caution. Current evidence more clearly supports reduced oxidative damage, fewer UV-induced DNA lesions, or improved cellular recovery in selected models; direct activation of defined DNA-repair pathways by MAAs is less established. The regulatory distinction is equally important. In the European Union, UV absorption alone does not make an ingredient an authorized UV filter: sunscreen filters are restricted to substances listed in Annex VI of the Cosmetics Regulation. MAAs should, therefore, currently be presented as photoprotective or antioxidant candidates, or as supporting actives, unless a specific compound obtains the relevant authorization [3].

### 3.3. Carotenoids, Chlorophyll Derivatives and Phycobiliproteins

Photosynthetic pigments are among the most recognizable algal ingredients, but their biological roles are broader than coloration and radical scavenging. Carotenoids such as beta-carotene, astaxanthin, fucoxanthin, lutein, zeaxanthin and alloxanthin can modulate redox-sensitive and inflammatory pathways; chlorophylls and their derivatives have distinctive photophysical properties and water-soluble phycobiliproteins such as phycocyanin and phycoerythrin combine chromophore activity with protein-mediated biological effects [85,86,87,88,89,90].

Astaxanthin is one of the most mature algal pigment examples. Preclinical studies support antioxidant and anti-inflammatory effects, including improvement of inflammatory phenotypes in atopic dermatitis models. Human oral-supplementation studies also suggest benefits for moisture and elasticity, although topical clinical evidence is much more limited [12,30,31]. Fucoxanthin suppressed NF-κB/MAPK-associated responses in macrophages [32]; its combination with rosmarinic acid modulated NLRP3-associated responses in UVB-exposed keratinocytes [33]. The combination result does not isolate the contribution of fucoxanthin alone. These mechanisms are relevant to photoaging and sensitive skin, but they still require confirmation with defined topical formulations.

Pigments also enable controlled photochemistry. Chlorophyll and pheophorbide derivatives can act as photosensitizers, and phycobiliprotein/chromophore systems can participate in photodynamic reactions. These properties support light-activated antimicrobial or anticancer strategies in which activity is triggered by illumination at an appropriate wavelength. A 2026 review summarizes the growing literature on microalgae as sources of photosensitizers for photodynamic therapy [46].

A dermocosmetic proof-of-concept particularly relevant to acne is the ethanolic extract of *Skeletonema marinoi*, enriched in pheophorbide a and related pigments. Under illumination, the extract showed strong activity against *Cutibacterium acnes*, *Staphylococcus aureus* and *Staphylococcus epidermidis*, including biofilm-related effects, while also revealing the critical need to manage phototoxicity toward keratinocytes [47]. This example illustrates both the opportunity and the design constraint of photoactivated dermocosmetics: spatial, spectral and dose control are essential to obtain microbial selectivity without damaging host tissue.

### 3.4. Peptides and Lipopeptides

Cyanobacteria are prolific producers of structurally diverse peptides, including linear peptides, cyclic peptides, depsipeptides and lipopeptides. They arise through both ribosomal pathways, often followed by extensive post-translational modification, and non-ribosomal peptide synthetases. Environmental competition has selected many of these compounds for antibacterial, antifungal, antiviral or cytotoxic functions, making cyanobacterial peptide chemistry substantially richer than the small number of peptides currently used in mainstream skincare [40,41,91].

Cyanobacterial peptide chemistry includes compounds such as aeruginazole A, brunsvicamides, lyngbyazothrins, pahayokolides, pitipeptolides, kawaguchipeptins, and laxaphycins. Reported activities include antibacterial, antifungal, antiviral, and cytotoxic effects. These molecules are valuable scaffolds for topical anti-infective discovery, but most remain far from a finished dermocosmetic ingredient. Activity against a laboratory pathogen is not enough: selectivity within a dysbiotic skin community, host-cell toxicity, hemolysis, sensitization, and stability must be assessed in parallel.

The most promising dermocosmetic direction may, therefore, be precision rather than maximal antimicrobial strength. Short peptides can potentially be engineered to suppress a pathobiont, interfere with quorum sensing or biofilm maturation, or modulate host defense while sparing commensals. Microalgae can also be developed as production platforms for recombinant antimicrobial peptides, an area that has advanced considerably by 2025 [42]. This converges with microbiome-aware skincare, where ecological selectivity is more valuable than indiscriminate sterilization.

Skin-directed peptide studies also support non-antimicrobial applications. A Chlorella-derived peptide reduced UVB-induced MMP-1-associated responses and restored procollagen-related signaling in human dermal fibroblasts, whereas nanoliposomal Spirulina protein hydrolysates accelerated full-thickness wound repair in vivo, supporting a broader role for algal peptides in matrix preservation and regeneration [44,45].

### 3.5. Lipids and Polyunsaturated Fatty Acids: Barrier Homeostasis and Inflammatory Tone

Microalgae are major biosynthetic sources of long-chain polyunsaturated fatty acids, glycolipids and unusual polar lipids. Their dermocosmetic relevance arises at two levels. First, lipids can directly support barrier physiology and influence epidermal differentiation. Second, omega-3 and related lipid mediators can modulate inflammatory signaling. The chemical form matters: free fatty acids, triglyceride oils, phospholipids and glycolipids have different oxidation sensitivities, sensory properties and delivery behavior.

Although algal oils are conceptually attractive, oxidation is a central formulation challenge. Highly unsaturated lipids require control of oxygen, light, metals and pro-oxidant interfaces and often benefit from encapsulation or antioxidant co-formulation. Recent microemulsion and nanoemulsion work is beginning to address this gap. For translation, efficacy should be separated from generic “omega-3” assumptions and demonstrated with the exact algal oil, concentration and topical formulation.

### 3.6. Scytonemin and Other Distinctive Secondary Metabolites

Scytonemin is a lipid-soluble extracellular pigment produced by selected cyanobacteria exposed to intense solar stress. Located in extracellular sheaths, it functions as a durable UV screen, particularly in the UVA range, and also exhibits antioxidant and anti-inflammatory activities. Its biosynthetic pathway and environmental regulation are increasingly understood, making it a plausible target for metabolic engineering [48,49,50].

Scytonemin illustrates the same regulatory issue as MAAs. Its broad UV absorption and photostability are scientifically attractive, but describing it as a “natural sunscreen” would imply a regulatory status it does not currently have. A more realistic near-term role is as an antioxidant or photodamage-supporting active, with UV-related effects used as mechanistic evidence rather than as an authorized sunscreen claim. Other cyanobacterial secondary metabolites may offer senomorphic, anti-inflammatory, or signaling functions, but their safety margins and target specificity remain essential prerequisites for cosmetic use.

A direct skin-inflammation study strengthens this mechanistic positioning: topical scytonemin reduced TPA-induced ear inflammation in mice and suppressed TNF-α, iNOS, and NF-κB-associated responses, supporting an anti-inflammatory role distinct from its optical UV-screening properties [51].

### 3.7. Chemically Defined Cyanobacterial Candidates

The cyanobacterial contribution becomes more apparent when the discussion moves from taxonomic extracts to chemically defined molecules. Several compounds already connect a specific structure to a skin-relevant mechanism, but they occupy very different translational levels. Sacran and C-phycocyanin have been tested directly on skin or in wound models; cyanobacterial MAAs and scytonemin provide defined photoprotective or anti-inflammatory chemotypes and specialized cyclic peptides offer precision scaffolds for controlling enzymes involved in extracellular-matrix remodeling. Keeping these levels separate is important: cyanobacterial chemical diversity is broad, but only a subset of molecules currently combines molecular definition with formulation- and skin-relevant evidence.

Sacran, the very-high-molecular-weight sulfated polysaccharide from *Aphanothece sacrum*, is one of the clearest cyanobacterial examples with direct human topical data. In a controlled study of healthy volunteers with a history of dry or inflamed skin, a sacran-containing serum did not significantly improve skin-surface water content or transepidermal water loss relative to placebo, but it improved several corneocyte-derived parameters, including corneocyte morphology, the IL-1RA/IL-1α ratio, and protein carbonylation [23]. Together with preclinical anti-inflammatory data [17], this supports a barrier-conditioning and stress-modulating rationale while also illustrating why hydration claims should not be inferred from polymer chemistry alone.

C-phycocyanin provides a complementary protein example: topical liposomal studies demonstrated cutaneous deposition and anti-inflammatory activity [37,85], C-phycocyanin-based wound models linked the protein to growth-factor-associated fibroblast and keratinocyte migration [38], and a 2025 study showed that topical phycocyanin accelerated wound closure in diabetic rats while increasing collagen and VEGF/Nrf2/HO-1-associated responses and reducing inflammatory mediators and MMP-9 [39]. These are not cosmetic efficacy trials, but they make phycocyanin a comparatively well-characterized cutaneous candidate whose translation will depend on purity, chromophore stability, proteolysis, and delivery.

At the small-molecule level, the cyanobacterial MAA series already provides useful structure-function examples: mycosporine-2-glycine has been linked to anti-glycation, collagenase-inhibitory, antioxidant, and anti-inflammatory effects [27,28], whereas the glycosylated 13-O-(β-galactosyl)-porphyra-334 from *Nostoc sphaericum* protected human keratinocytes from ultraviolet injury [29]. Scytonemin adds a chemically distinct UVA-absorbing and anti-inflammatory scaffold [48,49,50,51].

Particularly instructive is scytonemin monomer (ScyM), the last-step monomeric precursor of the dimeric pigment: after screening more than 4500 marine extracts, ScyM inhibited mushroom tyrosinase with an IC50 of 4.90 μM, compared with 11.31 μM for kojic acid, whereas dimeric scytonemin was inactive in the same tyrosinase screen [52]. This is a strong molecule-level discovery signal, but not yet evidence of depigmenting efficacy in human skin; melanocyte systems, reconstructed pigmented skin, topical exposure, and safety studies are still required.

Cyanobacterial cyclic peptides extend the molecular space toward enzyme-selective anti-remodeling strategies. Symplostatin 5 and related cyanobacterial peptides were characterized as potent, selective elastase inhibitors, with structural studies and epithelial-cell experiments also demonstrating cytoprotective and anti-inflammatory effects [92]. Tutuilamides A–C, unusual vinyl-chloride-containing cyclodepsipeptides isolated from marine cyanobacteria, provide a second structurally distinct family with potent elastase-inhibitory activity [93].

Because excessive elastase activity contributes to inflammatory tissue damage and extracellular-matrix loss, these compounds are mechanistically relevant to skin-aging and irritated-skin research. Their present evidence, however, is predominantly biochemical or non-cutaneous, and their potency and structural complexity demand careful assessment of cytotoxicity, selectivity, sensitization, stability, and topical exposure. They should, therefore, be considered precision natural-product scaffolds for dermocosmetic discovery rather than ready-to-use cosmetic ingredients.

**Figure 2:**
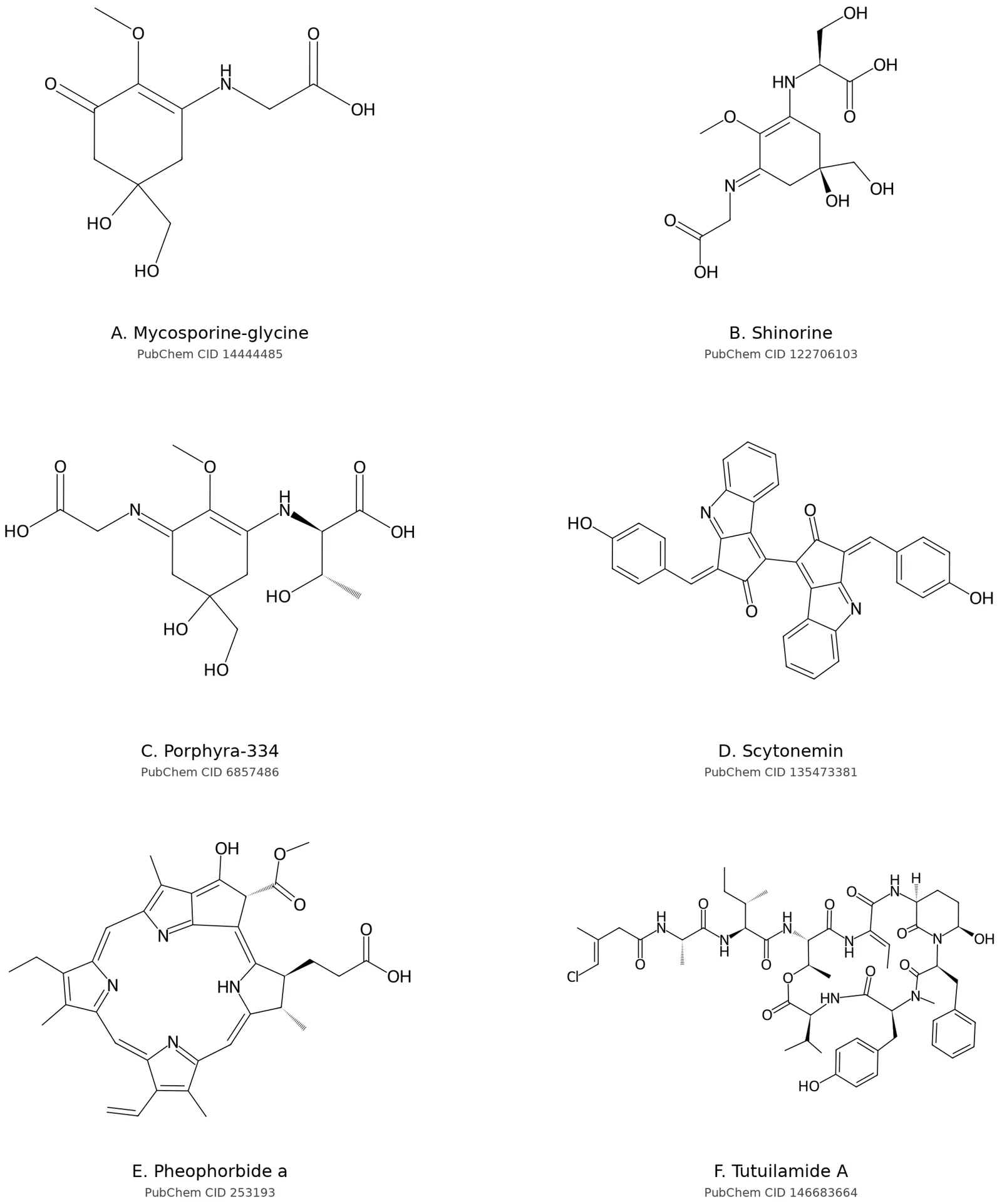
Representative molecular structures of microalgal and cyanobacterial metabolites. (**A**) mycosporine-glycine; (**B**) shinorine; (**C**) porphyra-334; (**D**) scytonemin; (**E**) pheophorbide a; (**F**) tutuilamide A. Structures were rendered from PubChem connection tables (CIDs printed in the panels; accessed 2 October 2026), using a chemical drawing engine. Stereochemistry is shown where specified in those records; unspecified centers are not assigned. The figure illustrates chemical diversity, not relative potency or clinical maturity [24,25,26,27,28,29,46,47,48,49,50,51,93].

Figure 2 illustrates chemically defined examples rather than treating class labels as structures. Mycosporine-glycine, shinorine, and porphyra-334 show variation around MAA chromophores; scytonemin provides a dimeric indolic pigment; pheophorbide a illustrates a tetrapyrrolic photosensitizer and tutuilamide A illustrates an Ahp-containing cyanobacterial cyclodepsipeptide. Their distinct substitution, charge, and conformational features explain why identity and composition must accompany biological activity [24,25,26,27,28,29,46,47,48,49,50,51,52,92,93].

### 3.8. Extracellular Vesicles and Nanoalgosomes: A Cell-Free Biological Delivery Layer

Extracellular vesicles (EVs) create a fundamentally different category from purified metabolites. These nanoscale lipid-bilayer particles naturally package proteins, lipids, nucleic acids and small metabolites and are designed by biology for intercellular transfer. Microalgal EVs, often referred to as nanoalgosomes in this context, offer a potentially scalable cell-free system that combines intrinsic cargo with a natural nanoscale carrier.

*Phaeodactylum tricornutum* EVs were reported to protect H_2_O_2_-stressed HaCaT cells and UV-exposed mouse skin, providing cellular and animal evidence (Levels D and C) [9]. *Tetraselmis chuii* nanoalgosomes reduced UVB-associated oxidative stress and senescence-related phenotypes and modulated melanogenesis in human skin-cell cultures (Level D) [10]. Human cellular origin must not be confused with testing in intact human skin, reconstructed tissue, or volunteers.

However, EV translation will require unusually rigorous characterization. Particle number alone is inadequate: source strain, culture conditions, isolation procedure, size distribution, surface markers, cargo composition, contaminating soluble molecules, storage stability and biological potency all need standardization. Regulatory classification is also unsettled, especially when claims approach tissue repair or disease modification. Nanoalgosomes may eventually become one of the field’s most distinctive innovations, but only if manufacturing science keeps pace with biological enthusiasm.

### 3.9. Structure-Function Relationships as the Basis for Ingredient Standardization

A recurring weakness across the field is the use of class names as though all members were functionally interchangeable. Figure 2 shows why this is problematic. MAAs share a compact cyclohexenone/cyclohexenimine chromophore but differ in amino-acid or amino-alcohol substitution and glycosylation, which alters absorption, polarity, redox behavior, and interaction with biological matrices. Scytonemin is a dimeric indolic-phenolic pigment, whereas its monomeric precursor has a substantially different molecular size and enzyme-interaction profile; the reported tyrosinase result for ScyM but not dimeric scytonemin is a direct example of structure-dependent activity [52]. Cyanobacterial Ahp-containing cyclodepsipeptides such as tutuilamide A add yet another scale of structural complexity, with macrocycle conformation and unusual residues determining protease recognition [92,93].

EPS require a different type of structure-function map because they are polydisperse macromolecules rather than single molecular entities. Molecular mass, branching, uronic-acid and sulfate substitution, monosaccharide sequence, and supramolecular conformation collectively control viscosity, film formation, charge, surface adsorption, and biological accessibility. Work on *Chlorella vulgaris* identified a highly branched arabino-rhamno-galactan with anti-inflammatory and anti-remodeling activity, while controlled depolymerization of microalgal EPS changed fibroblast responses: native high-molecular-weight material inhibited MMP-1 whereas lower-molecular-weight fractions increased collagen production [19,94]. Processing can, therefore, switch the dominant phenotype rather than merely improve penetration.

Small-molecule examples provide clearer molecular comparisons. Palythine, porphyra-334, shinorine, mycosporine-glycine, and mycosporine-2-glycine differ in ring oxidation state and substituent pattern, and glycosylation further expands the accessible physicochemical space. Palythine protected keratinocytes against several forms of UV-induced molecular damage, porphyra-334 reduced UV-induced apoptotic signaling, mycosporine-2-glycine inhibited glycation/collagenase and modulated inflammatory/oxidative responses, and glycosylated porphyra-334 protected human keratinocytes [25,26,27,28,29]. These observations justify reporting the identity and ratio of major MAAs rather than treating a mixed “MAA extract” as chemically self-explanatory.

The same logic applies to pigments, proteins, and peptides. A phycocyanin preparation is a chromoprotein whose purity, aggregation state, and chromophore integrity determine color and activity; pheophorbide a is a defined tetrapyrrolic photosensitizer whose useful antibacterial photochemistry is inseparable from host-cell phototoxicity and tutuilamide-type cyclodepsipeptides derive enzyme selectivity from a specific macrocyclic scaffold and unusual residues. High-value dermocosmetic development should, therefore, borrow the discipline of natural-product pharmacology: define the molecular or macromolecular material, identify critical structural attributes, establish an active range, and show that the manufacturing specification predicts biological potency.

**Figure 3:**
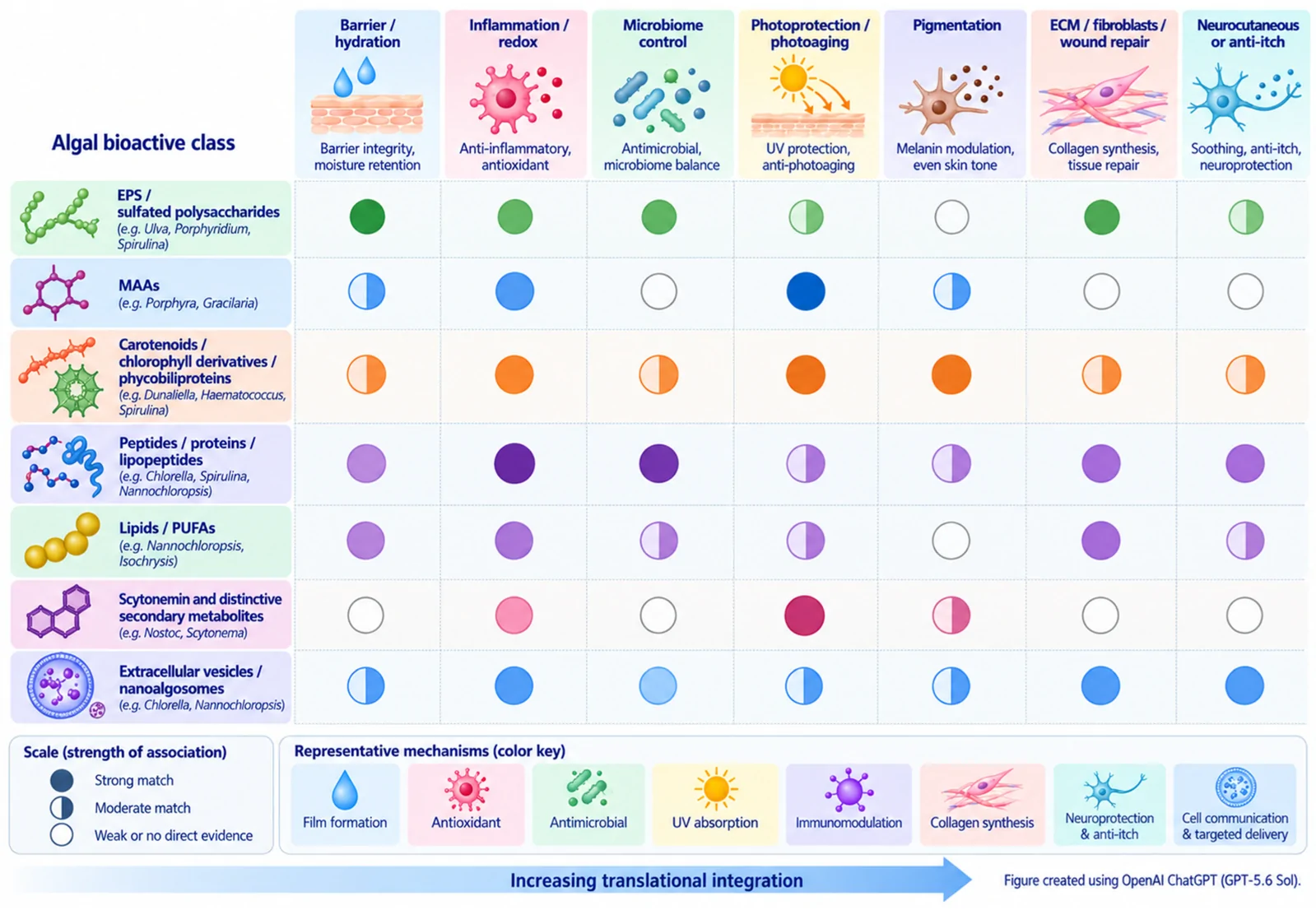
Matching algal bioactive classes to cutaneous targets and mechanisms. Exopolysaccharides, MAAs, pigments, peptides/proteins, lipids, distinctive secondary metabolites, and extracellular vesicles show different strengths of association with barrier support, inflammatory control, microbiome modulation, photoprotection, pigmentation, extracellular-matrix/wound repair, and neurocutaneous endpoints. Figure created using OpenAI ChatGPT (GPT-5.6 Sol). This is a conceptual map of candidate mechanisms, not an efficacy ranking or an evidence grade. Neurocutaneous and pollution-related links remain exploratory; supporting studies and limitations are discussed in Section 3, Section 4 and Section 5.

The principal correspondences between algal bioactive classes, cutaneous targets, and representative mechanisms are summarized in Figure 3.

## 4. Skin Mechanisms and Applications

### 4.1. Barrier Integrity, Hydration and Extracellular Matrix Support

Barrier support is probably the most defensible near-term dermocosmetic application of algal biotechnology. Deep penetration is not required for many relevant effects: a formulation can form an occlusive or semi-occlusive film, retain water, support surface lipids, reduce irritation, or modulate keratinocyte stress. EPS are particularly suitable because their physical properties are themselves functional. Even when largely retained at the surface, an EPS can reduce water loss, modify particle or microbial adhesion, and maintain a hydrated environment favorable to barrier recovery. Recent reviews of algal compounds for skin-barrier repair support this surface-first rationale [95].

For aging and post-procedural skin, matrix support adds a second level. Antioxidant and anti-inflammatory algal compounds can reduce pathways that drive metalloproteinase expression, while hydrated polymeric matrices can improve the mechanical microenvironment for re-epithelialization. Claims regarding collagen synthesis or wrinkle reduction should nevertheless be made only when confirmed in human-relevant models or clinical studies; cell-line increases in procollagen are useful mechanistic data, not final evidence.

### 4.2. Cutaneous Immunity and Inflammatory Pathways

Skin inflammation is not a single pathway and cannot be reduced to suppression of NF-κB in a macrophage assay. The cutaneous immune system integrates keratinocytes, Langerhans cells and other dendritic cells, resident and recruited T cells, macrophages, mast cells, innate lymphoid cells, fibroblasts, endothelial cells, and sensory neurons. Keratinocytes are active sentinels that release cytokines, chemokines and antimicrobial mediators after barrier damage or microbial sensing; antigen-presenting cells connect local danger signals to adaptive responses; mast-cell and neuronal mediators contribute to itch and neurogenic inflammation and fibroblasts participate in chronic inflammatory remodeling.

These compartments communicate through pattern-recognition receptors, NF-κB and MAPK pathways, inflammasomes, including NLRP3, eicosanoids, redox-sensitive transcription factors, and cytokine networks [1,2].

For the algal interventions considered here, pathway modulation is supported at different levels. Fucoxanthin suppressed inflammatory NF-κB/MAPK-associated outputs in LPS-stimulated macrophages (Level D), whereas the fucoxanthin–rosmarinic acid combination was studied in UVB-exposed keratinocytes and cannot establish a fucoxanthin-only effect [32,33]. Topical scytonemin reduced TPA-induced mouse-ear inflammation (Level C) [51], and phycocyanin was associated with Nrf2/HO-1, inflammatory, and repair responses in diabetic rats (Level C) [39]. Changes in cytokines or pathway proteins establish an association unless supported by target inhibition, genetic perturbation, or rescue experiments. They should not be interpreted as proof that a compound directly controls an entire cutaneous immune network.

### 4.3. Skin Microbiota and Selective Intervention

The skin microbiome is site-specific and functionally heterogeneous. *Cutibacterium acnes* is a dominant commensal of sebaceous sites, but particular strain backgrounds and host contexts are associated with acne; *Staphylococcus epidermidis* is commonly health-associated and can contribute to colonization resistance, yet selected strains may become opportunistic; *Staphylococcus aureus* is a major pathobiont in atopic dermatitis and damaged skin. Corynebacterium and other genera also contribute to community structure. Consequently, a reduction in total bacterial burden is not a meaningful microbiome endpoint by itself. The desirable outcome is preservation or restoration of a functional community while limiting virulence, overgrowth, adhesion, or biofilm behavior of context-relevant pathobionts [96,97,98].

Microalgal and cyanobacterial compounds can influence this ecology through at least four separable mechanisms: selective antimicrobial activity, anti-adhesion, interference with quorum/biofilm processes, and indirect modification of the host surface through hydration, pH, lipids, or inflammatory tone. EPS are particularly attractive when they reduce adhesion without broad bactericidal pressure, whereas peptides offer the opposite strategy of high-potency molecular recognition. The two approaches require different assays: an anti-adhesive polymer should be tested on relevant surfaces and polymicrobial consortia, while a peptide should be profiled for strain-level antimicrobial selectivity, keratinocyte toxicity, hemolysis, and proteolytic stability.

The experimental standard should progress from single isolates to defined consortia or colonized reconstructed skin, then to human studies that combine microbial and host endpoints. Useful readouts include strain-resolved abundance, alpha/beta diversity, biofilm architecture, barrier recovery, inflammatory markers, sebum or lipid changes, and clinical phenotype. The 2025 vehicle-controlled Spiralin study is notable because it tested a defined Arthrospira-derived topical preparation in a small controlled human study; the microbiome result and clinical trends require separate interpretation [99]. Such studies illustrate the level of evidence needed before “microbiome balancing” can be treated as more than a marketing phrase.

### 4.4. Photoprotection, Photoaging and DNA-Damage Limitation

Photoprotection is one of the clearest areas where algal evolution and human skin biology intersect. MAAs and scytonemin absorb harmful wavelengths; carotenoids quench excited states and modulate oxidative signaling; EPS can provide film-forming and hydration support and antioxidant systems can reduce downstream inflammatory amplification. A rational formulation could, therefore, combine optical attenuation with biological resilience rather than relying on a single mechanism.

Nevertheless, photoprotection must be divided into regulatory and biological layers. A formulation sold as a sunscreen requires authorized UV filters and validated SPF/UVA protection according to the relevant jurisdiction. A non-filter algal active may still reduce oxidative stress or photoaging biomarkers, but this is a supportive role. Such distinction is particularly important when discussing MAAs or scytonemin, because attractive absorption spectra can easily lead to language that exceeds current authorization [3].

Future studies should move beyond short-term ROS assays. Useful endpoints include cyclobutane pyrimidine dimers, 8-oxo-dG, mitochondrial function, MMP expression, inflammatory cytokines, pigmentation signaling and long-term recovery after repeated UVA/UVB or visible-light exposure. Reconstructed pigmented skin and repeated-dose protocols are especially valuable for assessing whether a candidate provides durable biological protection rather than transient radical scavenging.

### 4.5. Neuroimmune Stress, Senescence and Pollution

The skin-brain axis and cellular senescence are increasingly relevant to sensitive-skin and healthy-aging research, but algal evidence remains less mature than for barrier or photoprotection. Sensory nerves, keratinocyte-neuron communication, neuropeptides, transient receptor potential channels, mast cells, mitochondrial stress, and the senescence-associated secretory phenotype form an interconnected neuroimmune-stress network. Generic antioxidant or anti-inflammatory activity cannot be assumed to translate into reduced itch, stinging, pollution resilience, or senescence modification.

At present, carotenoids, MAAs, phycobiliproteins, and selected polysaccharides are better described as stress-resilience or senomorphic candidates than as proven neurocosmetic or senolytic agents. Relevant development models include sensory-neuron/keratinocyte co-cultures, chronic low-dose pollutant plus UV exposure, mitochondrial-function assays, and explicit distinction between senolysis and suppression of SASP-associated outputs. Human sensory endpoints will be essential for itch or stinging claims [2,14,15].

### 4.6. Wound Healing, Regenerative Interfaces and Photoactivated Antimicrobial Systems

Wound healing is where algal bioactivity and advanced delivery converge most visibly. EPS can provide hydrated matrices and bioadhesive interfaces; pigments and peptides can contribute antimicrobial or anti-inflammatory effects and microalgal metabolites may influence fibroblast migration, extracellular matrix deposition and re-epithelialization. Nostoc-derived polysaccharides, for example, have been investigated for barrier repair and wound-associated effects [18].

Wound-healing research has already moved well beyond simple extracts. A 2026 review describes hydrogels, scaffolds, sutures, microneedles, and microrobots that use photosynthetic oxygen production to counter wound hypoxia [100]. A second 2026 review similarly traces the transition from bioactive algal metabolites to living photosynthetic scaffolds that combine oxygenation, antimicrobial activity, and regenerative support [101]. These technologies are biomedical rather than conventional cosmetics, but they provide a clear innovation path for post-procedural and regenerative skin interfaces.

Photoactivation offers temporal control: a pigment-containing formulation can remain relatively inactive until exposed to a selected wavelength, after which reactive species are generated locally. This principle is valuable for acne-associated microorganisms and biofilms because illumination can spatially confine activity and reduce the need for continuously high antimicrobial concentrations.

However, once the mechanism is explicitly cytotoxic and intended to treat infection, neoplasia or another disease, the system moves toward photodynamic therapy and, therefore, outside ordinary cosmetic positioning. The distinction should be preserved even in a forward-looking review. Microalgal photosensitizers are valuable because they inspire safer and more sustainable photodynamic systems, but tumor ablation or postoperative melanoma control must be discussed as biomedical proof-of-concept, not as dermocosmetic functionality [46,54].

### 4.7. Human Evidence and Claim-Relevant Endpoints

Human evidence remains uneven across algal actives. Astaxanthin has the broadest clinical literature, although many studies concern oral supplementation rather than a defined topical microalgal formulation. One systematic review identified eleven clinical studies, including six randomized double-blind placebo-controlled trials, with signals for improved moisture, texture, wrinkles, and protection from UV-related damage. Confidence was limited by small sample sizes, a strong concentration of healthy Japanese women, and commercial sponsorship [13].

Earlier oral and combined oral/topical studies also reported improvements in selected wrinkle, elasticity, and transepidermal water loss endpoints, again in modest samples [34]. These results support biological plausibility but cannot be generalized to every astaxanthin-containing cosmetic.

Sulfated polysaccharides from red microalgae reduced irritant-associated erythema in human testing and were also examined in neutrophil assays [16]. Sacran provides a distinct controlled topical example: corneocyte-derived parameters improved, whereas water content and transepidermal water loss did not significantly differ from placebo [23]. Surface activity is, therefore, plausible without deep penetration, but the supported endpoint must be reported rather than replaced by a generic moisturization claim.

A double-blind, intraindividual vehicle-controlled study tested Spiralin gel on paired atopic-dermatitis lesions in 20 participants for four weeks [99]. The Shannon index increased by 16.7% at active-treated sites (*p* < 0.01 versus baseline); this within-site result is not equivalent to demonstrated between-treatment superiority.

The reductions in *Staphylococcus aureus* abundance were 25.5% with active gel and 9.4% with vehicle, but the difference was not significant. Clinical changes were exploratory trends. This is Level A by study design, with low precision and limited efficacy conclusions; it does not establish general treatment efficacy for atopic dermatitis.

For MAAs, phycocyanin, nanoalgosomes, and most peptide candidates, controlled clinical evidence is still absent or insufficient. The key question is, therefore, what experiment should come next. MAA formulations need human or ex vivo photobiology with validated DNA-damage and erythema endpoints. Nanoalgosomes require standardized EV preparations, defined doses, sensitization testing, and controlled studies of finished formulations. Microbiome claims require strain-resolved ecological measurements linked to barrier and inflammatory outcomes. Framing the evidence this way makes the translational gap explicit rather than simply describing each technology as “promising.”

Table 4 summarizes the human-relevant and near-human model systems most useful for substantiating microalgal and cyanobacterial skin claims.

## 5. Formulation, Delivery and Translational Validation

### 5.1. Matching Molecular Class, Target Depth and Exposure

Algal molecules span extreme physicochemical diversity. EPS are large and hydrophilic; MAAs are small and water-soluble; carotenoids are highly lipophilic and oxidation-prone; phycobiliproteins are large, fragile proteins; peptides can be rapidly degraded and extracellular vesicles are colloidal biological particles. No single formulation platform can solve all of these constraints. Delivery must, therefore, be matched to molecular class and intended skin compartment.

For many dermocosmetic endpoints, deep transdermal penetration is neither necessary nor desirable. Surface-retained EPS may be optimal for hydration and anti-adhesion; a photoprotective MAA may function within the upper epidermal environment; lipophilic carotenoids may require delivery into viable epidermal membranes and photoactivated antimicrobial agents should be localized where microorganisms reside. Rational formulation begins by defining the target depth and residence time rather than maximizing penetration indiscriminately.

**Figure 4:**
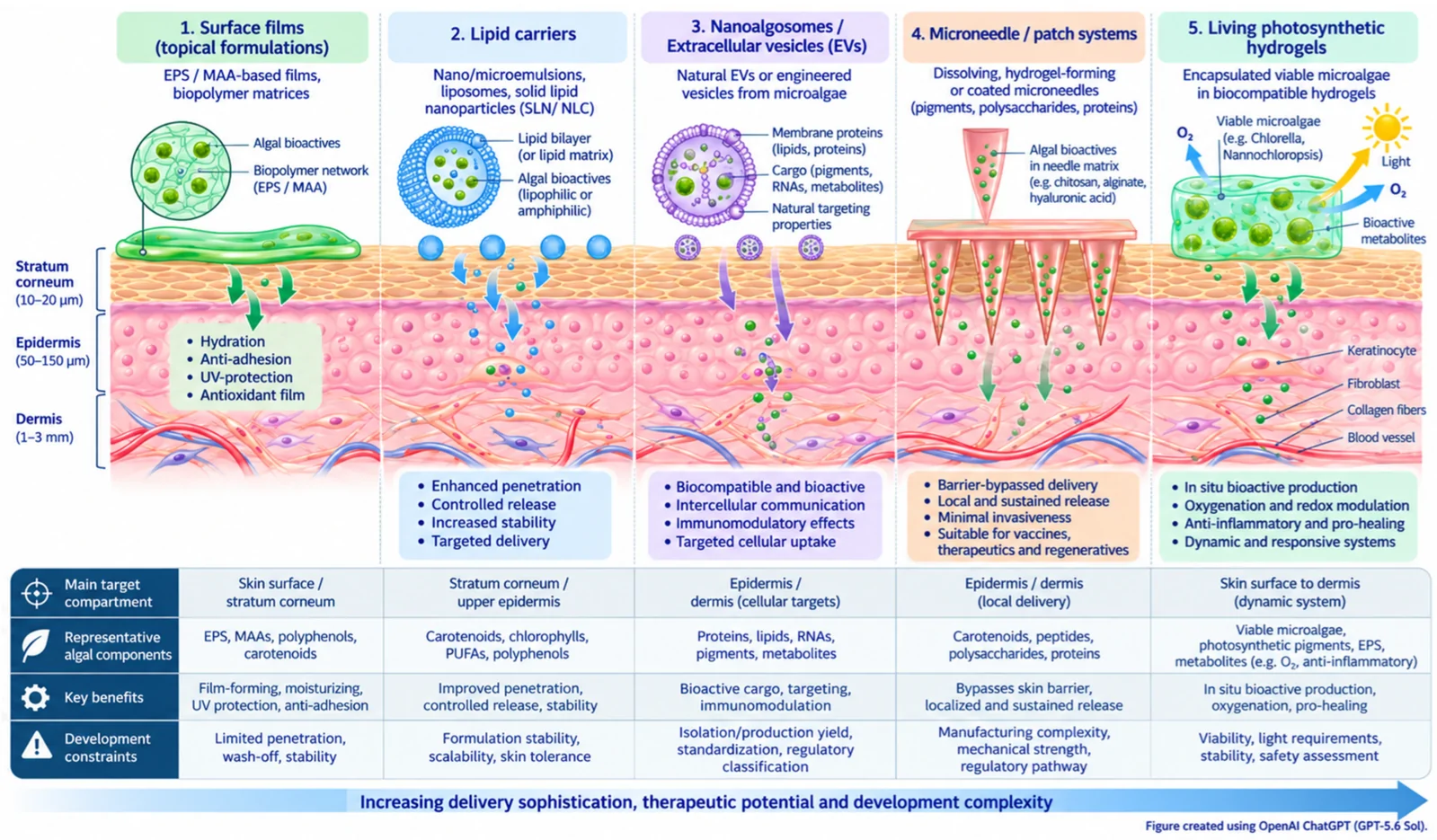
Conceptual delivery architectures for algal skin technologies. Surface films, lipid carriers, extracellular vesicles, microneedle/patch systems, and living hydrogels address progressively different tissue compartments and development constraints. Figure created using OpenAI ChatGPT (GPT-5.6 Sol).

The principal delivery architectures and the skin compartments they are intended to address are illustrated in Figure 4.

### 5.2. Conventional, Lipid and Polymeric Carriers

Conventional emulsions remain useful because they are scalable and familiar, but nanostructured carriers can improve protection and dispersion of unstable pigments and lipids. Liposomes, nanoemulsions, solid lipid nanoparticles and nanostructured lipid carriers can shield carotenoids from oxygen and light, improve apparent solubility and modulate epidermal deposition. The improvement of liposomal astaxanthin over non-encapsulated material in an inflammatory skin model illustrates how formulation can alter biological performance [31].

Encapsulation performance alone does not establish clinical efficacy. Particle size, interfacial composition, release kinetics, occlusivity, skin deposition and sensory properties all affect the finished formulation, and excessive penetration may increase irritation. Stability under realistic packaging, temperature and light exposure is as important as initial encapsulation efficiency.

Hydrogels and polymeric films are particularly compatible with algal polysaccharides and regenerative applications. They maintain hydration, can absorb exudate, protect labile payloads and create prolonged contact with the skin. Composite systems can combine a structural polymer with pigments, peptides, EVs or conventional drugs. Nanofibers add high surface area and tunable release but can be more demanding to manufacture reproducibly.

An important trend is multifunctionality: the matrix is no longer an inert carrier but contributes to biological performance through hydration, oxygen transfer, adhesion or responsiveness to pH and enzymes. This is conceptually aligned with microalgal EPS, which can act simultaneously as rheological modifiers and bioactive interfaces.

### 5.3. Advanced Delivery: Microneedles, Extracellular Vesicles and Living Photosynthetic Materials

Microneedles bypass the stratum corneum with minimal invasiveness and can deliver hydrophilic macromolecules or fragile biological payloads into viable skin. For ordinary cosmetics, this raises classification issues, but as a biomedical technology it is highly relevant to the future of skin delivery. Dissolving, hydrogel-forming and hollow microneedles can be coupled to light, pH, ROS or enzymatic triggers to produce spatially and temporally controlled release.

A photosynthesis-powered dual-layer pneumatic microneedle patch was reported for deep wounds in 2025. It combined oxygen-producing cyanobacteria with sesamin and used photosynthetically generated gas to support delivery beyond 1000 μm, addressing both hypoxia and depth limitations [55]. This biomedical proof of concept illustrates how photosynthesis can contribute to both the mechanical and biochemical functions of a delivery device.

Nanoalgosomes challenge the conventional distinction between active and carrier because the vesicle is both. Their lipid membrane can protect endogenous cargo, facilitate cellular uptake and potentially be engineered or loaded with additional molecules. The Phaeodactylum and Tetraselmis studies cited above show that skin cells can interact with these particles and that biologically relevant effects are measurable [9,10].

Future work should compare nanoalgosomes against matched soluble extracts and artificial liposomes. Such controls are critical to determine whether the advantage arises from vesicular delivery, unique cargo, or simply co-purified metabolites. Scalable purification—likely involving combinations of tangential-flow filtration, size-exclusion or other chromatographic methods—must preserve biological potency while minimizing batch variability.

Living microalgae or cyanobacteria embedded in biomaterials provide a further delivery modality. Under illumination, these cells can generate oxygen continuously at the treatment site, potentially addressing local wound hypoxia. The material can be designed to confine the cells while allowing diffusion of gases or secreted factors.

An earlier photosynthetic alginate hydrogel containing *Chlamydomonas reinhardtii* demonstrated light-dependent oxygen release and was additionally evaluated on healthy human skin in 20 volunteers without detectable irritation under the reported protocol, providing an unusual bridge between biomaterial proof-of-concept and human tolerability [11].

A 2024 living photosynthetic microneedle system combined in situ oxygenation with postsurgical melanoma therapy, demonstrating the biomedical potential of microalgae-containing patches [54]. In 2025, a photo-crosslinked microalgae-containing hydrogel with antimicrobial co-therapy accelerated diabetic wound closure in vivo, while the photosynthetic pneumatic patch described above targeted deep wounds [55,56]. By 2026, reviews were already describing a broader landscape of photosynthetic living scaffolds, genetically engineered microalgae and 3D-bioprinted systems [100,101].

These platforms raise containment, microbiological quality, shelf-life, illumination and host-interaction questions that differ from those of conventional skincare. Their relevance to future skin interfaces lies in the ability to supply a function continuously and locally, rather than release only a finite dose of an ingredient. They should, therefore, be evaluated as living biomedical systems, with the corresponding safety and manufacturing requirements.

### 5.4. Formulation Constraints and Target Depth

Water-soluble polysaccharides and MAAs appear formulation-friendly because they are compatible with aqueous phases, but their challenges are different. EPS can create excessive viscosity, stringiness, precipitation with cationic ingredients or preservative-binding effects; high electrolyte content can also alter rheology. MAAs are smaller but require protection from pH-dependent degradation and must remain spectrally stable under light and storage conditions. For both classes, in-formula activity should be tested after accelerated stability rather than inferred from freshly prepared solutions.

Carotenoids and PUFA-rich lipids are dominated by oxidation and solubility constraints. Oxygen, heat, light and transition metals can drive isomerization or peroxidation, making packaging and antioxidant systems part of the active design. Nanoemulsions, lipid nanoparticles, cyclodextrin-based approaches and liposomes can improve dispersibility and protect labile actives, but carrier composition can also determine skin deposition and irritation. The most informative studies, therefore, compare free and delivered active at equal dose and measure chemical stability and biological effect in parallel [31,35,36].

Proteins and peptides face enzymatic degradation, aggregation and limited barrier penetration. The classic phycocyanin liposome study is instructive because the chromoprotein remained predominantly in the stratum corneum rather than permeating through full-thickness skin, yet topical anti-inflammatory performance was improved [37]. For dermocosmetics, this is not necessarily a failure: many targets are epidermal or surface-associated. Delivery should be optimized for the intended compartment, not for maximal systemic penetration.

EVs introduce an additional requirement: the carrier is also the active. Formulation excipients must preserve membrane integrity and cargo function, and assays must distinguish intact EV effects from co-isolated soluble molecules. Freeze-thaw, drying, preservatives and surfactants may alter EV potency. Development, therefore, needs stability-indicating particle metrics together with a biological potency assay, analogous to the quality-by-design principles used for more complex biologics.

A simple design rule is to define the target depth before choosing the carrier. Hydration, anti-adhesion, and many microbiome effects occur at or near the stratum corneum, so aggressive penetration enhancement is unnecessary. Keratinocyte signaling and pigmentation require access to the viable epidermis, whereas fibroblast and extracellular-matrix targets require dermal exposure, which is much harder to achieve through intact skin. In wounds or postsurgical sites, the barrier is disrupted, allowing larger proteins, EVs, and living materials to interact more directly with tissue.

This target-depth map prevents a common formulation error: assuming that deeper penetration is always better. For a cosmetic, excessive permeation can increase systemic exposure and safety requirements without improving efficacy. Conversely, claims about dermal collagen remodeling are difficult to support if the active remains exclusively on the surface. Combining tape stripping, confocal imaging, Franz-cell studies and reconstructed-skin pharmacodynamics can establish whether the exposure profile is consistent with the intended mechanism.

Representative links between delivery strategies and target skin compartments are summarized in Table 5.

Selected recent examples of emerging microalgal and cyanobacterial skin-delivery platforms are compiled in Table 6.

### 5.5. Matching Validation Models to the Intended Claim

The algal literature is rich in activity but uneven in translational depth. A DPPH or ABTS assay can document chemical radical-scavenging capacity; a macrophage assay can reveal anti-inflammatory signaling; a cancer cell line can identify cytotoxic potential. None of these alone demonstrates a dermocosmetic effect. A practical evidence hierarchy should progress from physicochemical characterization to cutaneous cell models, reconstructed skin, ex vivo human skin, relevant in vivo models and ultimately controlled human studies.

The hierarchy should also reflect the claim. Hydration can be substantiated with corneometry and transepidermal water loss; barrier repair requires repeated measurements and ideally lipid/structural endpoints; anti-redness claims need standardized imaging or colorimetry; photoaging support can combine clinical imaging with validated biomarkers; microbiome claims require sequencing or targeted quantitative methods and ecological interpretation. The closer a claim approaches disease treatment, the stronger the regulatory and clinical requirements become.

**Figure 5:**
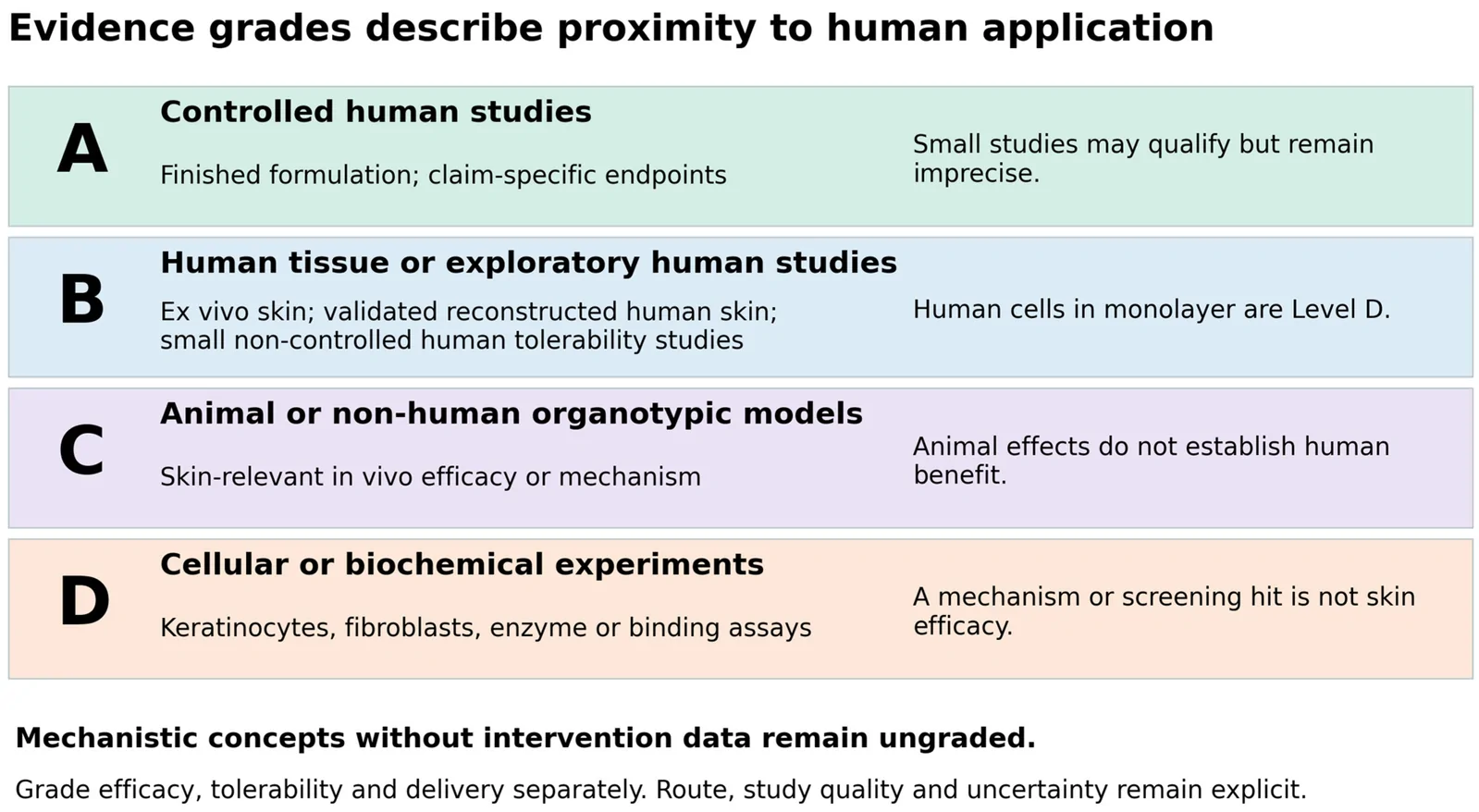
Operational evidence grades used in this review, aligned with Section 1.1 and Table 1, Table 4, Table 5 and Table 6. Controlled human studies are Level A; uncontrolled human studies and human-tissue/reconstructed-skin studies are Level B; animal and non-human organotypic studies are Level C; cellular and biochemical studies are Level D. Mechanistic concepts remain ungraded. Grade describes model proximity, not study quality or certainty, and efficacy, delivery, and tolerability are assessed separately. Original schematic prepared for this review.

A practical evidence cascade aligned with translational claim substantiation is presented in Figure 5.

### 5.6. Human-Relevant Models, Omics and Data-Assisted Development

Reconstructed human epidermis already has regulatory acceptance for several safety applications and is highly useful for irritation, barrier and photodamage studies. Full-thickness equivalents add fibroblasts and extracellular matrix, enabling more informative studies of inflammation, remodeling and wound repair. Skin organoids further expand cellular complexity and developmental architecture, although standardization and throughput remain challenges.

A 2025 review in Acta Biomaterialia emphasized the progression from two-dimensional cultures to skin equivalents and organoids for dermatology and drug testing [102]. For algal ingredients, these models can answer practical questions that simple monolayers cannot: whether a high-molecular-weight EPS remains surface-localized, whether a photosensitizer damages keratinocytes at antibacterial light doses, whether an EV crosses the barrier, or whether a pigment affects melanocyte-keratinocyte signaling in a tissue context.

Skin-on-chip systems add flow, mechanical cues, vascular-like compartments and controlled molecular transport, enabling a more dynamic representation of human skin. In 2026, a major review of skin microphysiological systems highlighted 3D bioprinting, organoids and skin-on-chip as key nonclinical technologies, while another review focused specifically on dynamic biomimicry for dermatology, drug screening and cosmetic evaluation [104,105].

These platforms are particularly relevant to next-generation algal systems. Microbiome-integrated chips could test selective antimicrobial effects; vascularized chips could distinguish local from systemic exposure; photosynthetic biomaterials could be assessed under controlled oxygen and light gradients and repeated-dose models could capture cumulative irritation or adaptation. Standardized interlaboratory performance remains a prerequisite for routine industrial use.

Metabolomics and high-resolution mass spectrometry are transforming algal extracts from black boxes into chemically resolved mixtures. Transcriptomics and proteomics can then reveal how skin cells respond, while genome mining can connect metabolites to biosynthetic pathways and guide strain engineering. Multi-omics is especially important for complex EPS and EV preparations, where activity may arise from coordinated rather than single-compound effects.

AI may help integrate these data by ranking candidates, identifying formulation incompatibilities or stratifying users. Its reliability is limited by small, heterogeneous cosmetic datasets. Prospective experimental validation, transparent feature reporting and predefined performance metrics are, therefore, needed to establish its contribution to scientific substantiation [107,108].

A credible translational package for a new algal active should combine taxonomic and manufacturing traceability, quantitative chemical fingerprinting, contaminant control, formulation stability, exposure-relevant cutaneous pharmacology, and safety. It should also show that the selected human-relevant endpoint matches the intended claim and that the finished formulation retains the activity observed with the isolated ingredient. This is more demanding than a conventional activity screen, but it provides a shorter and more defensible route from promising biology to a reproducible product.

Clinical studies should follow the same mechanistic chain. A barrier product should combine corneometry and transepidermal water loss (TEWL) with lipid or tight-junction biomarkers where feasible. A microbiome product should prespecify ecological endpoints and link them to clinical or barrier changes. A photoaging product should include standardized imaging or biophysical endpoints and sufficient exposure duration. Maintaining this continuity from in vitro experiments to human studies helps determine whether the proposed mechanism produces a measurable skin benefit.

## 6. Manufacturing, Sustainability and Regulatory Boundaries

### 6.1. Quality, Safety and Sustainability by Design

Manufacturing claims should be evaluated at the level of a defined process. Microalgae can reduce dependence on arable land and can be coupled to CO2 or nutrient-recovery streams, but closed photobioreactors, artificial lighting, harvesting, drying, solvent recovery, and purification can also carry substantial energy and material burdens.

The same process choices influence biological quality: oxidation, shear, heat, contaminants, and batch variability can change potency. Sustainability, safety, and quality-by-design should, therefore, be assessed together rather than as separate end-stage topics. Quantitative comparisons must use an explicit functional unit, such as one kilogram of standardized ingredient or one batch of stable finished formulation, rather than comparing unlike extract masses.

Practical priorities include wet-biomass processing where feasible, extracellular-product recovery, fraction cascades and coproduct valorization, solvent and energy accounting, contaminant limits, and critical quality attributes tied to potency. For complex fractions, batch comparability requires both analytical fingerprints and orthogonal biological assays. For photoactive or membrane-active compounds, phototoxicity or host-cell selectivity must be part of the same specification used to justify efficacy.

### 6.2. Regulatory Boundaries: Cosmetics, UV Filters and Advanced Delivery Systems

In the European Union, cosmetic products are governed by Regulation (EC) No 1223/2009, and claims must satisfy common criteria, including truthfulness and adequate evidential support under Commission Regulation (EU) No 655/2013 [3,6]. Natural or marine origin does not alter classification. In particular, MAAs, scytonemin, and other novel UV-absorbing molecules are not authorized sunscreen filters simply because they absorb UV; use as a UV filter requires inclusion in Annex VI. They may instead be developed for appropriately substantiated antioxidant or photoaging-support claims.

In the United States, sunscreens are regulated as over-the-counter drugs, so photoprotective actives follow a different route from ordinary cosmetics. The framework is still evolving. In June 2026, the U.S. Food and Drug Administration added bemotrizinol to the permitted sunscreen active ingredients, the first new active added to the OTC monograph in roughly two decades [109]. For algal photoprotectants, this is both encouraging and instructive: new filters can reach the market, but only after meeting a high evidentiary threshold.

### 6.3. Techno-Economic Interpretation and Translation to a Finished Formulation

Potential sources of commercial differentiation include proprietary strains, analytically standardized molecular fractions, clinically substantiated functions, and delivery systems with demonstrated performance. Nanoalgosomes, functional EPS, metabolically programmed photoprotective fractions, and selective peptide scaffolds are examples. Additional biological complexity is justified only if the resulting benefit offsets manufacturing, stability, safety, and regulatory costs.

The astaxanthin comparison in Table 3 illustrates why recovery technology changes economic performance, but also why published process costs cannot be transferred directly to a dermocosmetic. Its annual costs refer to a modeled production facility and its original assumptions, not to current quotations, clinical development, or a finished cream [67]. A process for another species, molecular class, grade, or scale requires its own mass balance and cost basis.

For a defined ingredient, a useful cost model divides annualized capital charges plus operating expenses, quality-control costs, and waste treatment, minus defensible coproduct credits, by the annual mass that meets the release specification. Sensitivity analysis should vary culture productivity, product fraction, recovery at each operation, energy price, solvent reuse, labor, downtime, and batch rejection. Credits should require a characterized coproduct and a credible outlet.

Costs of discovery, analytical reference standards, toxicology, formulation development, packaging, and human substantiation must then be added separately according to product classification and development stage.

Delivery imposes a further economic denominator: the stable, active dose reaching the intended site. As an illustrative calculation, if only half of an initially incorporated active remains within specification at shelf-life, twice the starting amount would be required to retain the same nominal active mass, before accounting for delivery losses or changes in safety. This is not a dosing recommendation.

For EVs and living materials, release testing must additionally quantify particle/cargo integrity or viable-cell function, storage losses, and light-dependent output. The cited studies do not provide a comparable, validated end-to-end cost per effective human dose for these emerging systems [9,10,11,53,54,55,56].

Thus, the most defensible current strategy is to select the least burdensome process that delivers the required identity, potency, stability, safety, and exposure, and to demonstrate any advantage of a more complex carrier against an appropriate simpler comparator. Table 6 distinguishes quantitative human observations from cellular and preclinical delivery results; a high encapsulation efficiency or a favorable ingredient-production estimate does not establish clinical or commercial superiority.

**Figure 6:**
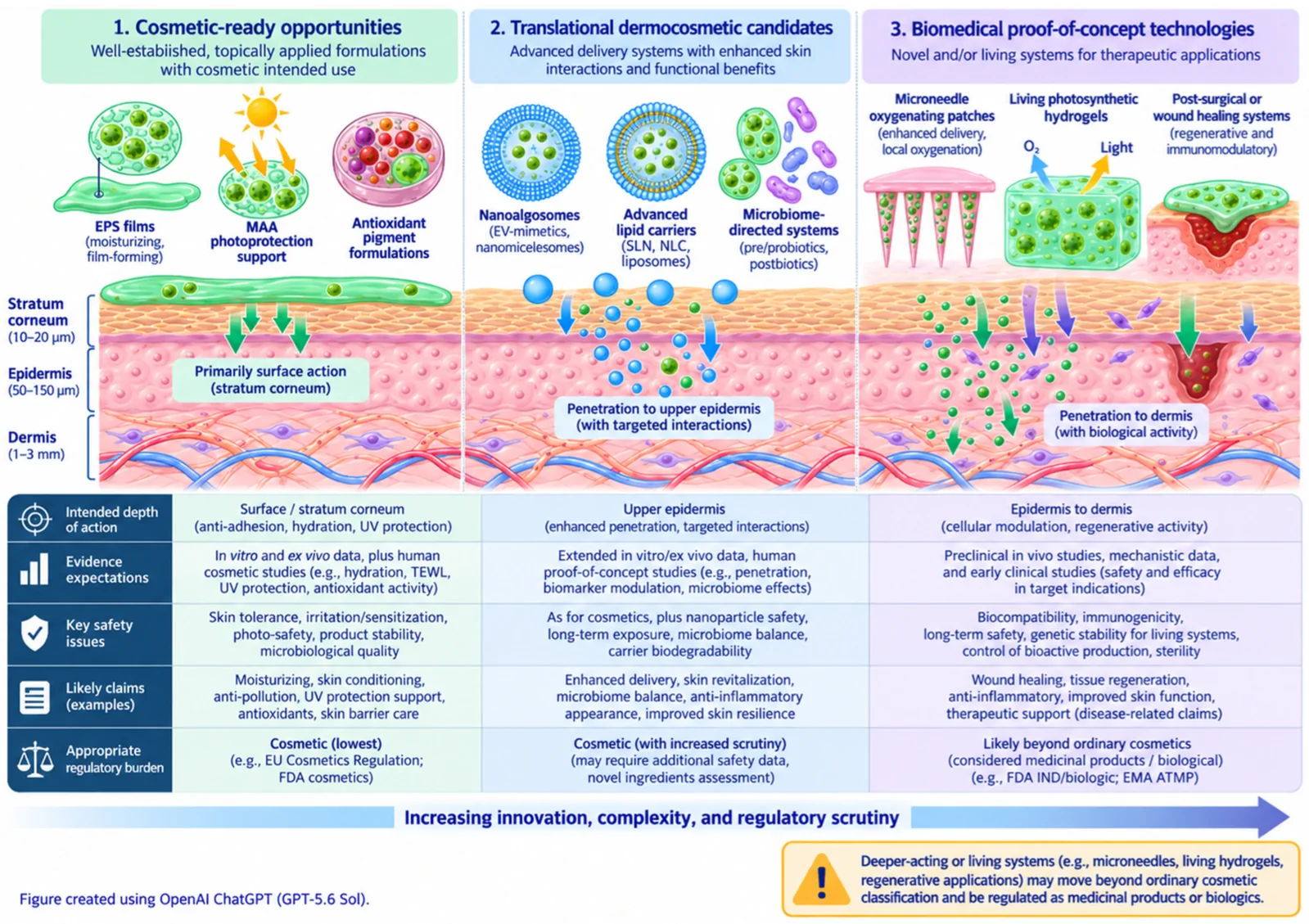
Development positioning and regulatory boundaries of algal skin technologies. Cosmetic-ready opportunities, more advanced translational dermocosmetic candidates, and biomedical proof-of-concept systems differ in intended depth of action, evidence expectations, safety requirements, and probable regulatory burden. Figure created using OpenAI ChatGPT (GPT-5.6 Sol). The depicted tiers are development scenarios, not legal determinations: penetration depth alone does not determine classification. MAAs are not authorized UV filters by virtue of UV absorption, and disease-treatment or wound-healing claims require the appropriate regulatory route.

The resulting development positioning and associated regulatory boundaries are summarized in Figure 6.

## 7. Research Gaps and Roadmap to 2030

The main research gap is no longer a lack of positive antioxidant assays. It is the lack of hypothesis-driven skin biology. A candidate should advance because it changes a defined cutaneous process at a plausible topical exposure, and the strain, cultivation conditions, and chemical fingerprint should be reported as part of the active-material definition. Microbiome claims require the same discipline: activity against a pathobiont matters only when selectivity, community resilience, and host responses are considered. Photoactive systems add another requirement, because microbial or tumor phototoxicity must be interpreted together with host-cell phototoxicity under realistic illumination.

The field will also benefit from exploiting functions that are genuinely distinctive to photosynthetic microorganisms. Nanoalgosomes provide naturally assembled multi-cargo nanocarriers, whereas living microalgae or cyanobacteria can generate oxygen locally. Human-relevant reconstructed skin, organoids and microphysiological systems offer the appropriate bridge for testing these technologies before costly clinical studies, while controlled human trials should use standardized instrumental endpoints and adequate exposure duration.

Sustainability and regulation should, therefore, be treated as design variables rather than end-stage checks. Energy-intensive drying or purification can erode the environmental advantage of photosynthetic production, and an intended claim that implies drug action or UV-filter function can change the development route entirely. The most credible product concepts will emerge where biological function, delivery, manufacturability, evidence and legal positioning are aligned from the outset.

### Prospective Development Pathways

The most plausible near-term advances are standardized EPS for barrier/microbiome-supportive interfaces, more stable MAA and pigment formulations, and algal EV ingredients supported by reconstructed-skin and early clinical evidence. These products can remain within familiar topical formats while offering better mechanistic substantiation than current extract-based cosmetics.

A second wave will likely combine multiple functions in one engineered interface: an EPS hydrogel that controls hydration and adhesion, a nanoencapsulated pigment that responds to light, a peptide that selectively limits a pathobiont, or a vesicle system that delivers a complex stress-response cargo. The emphasis will shift from ingredient lists to spatially and temporally coordinated functions.

A third wave, more biomedical than cosmetic, will involve living photosynthetic materials, gene-engineered microalgae, 3D-bioprinted constructs and responsive microneedle systems. Their relevance to chronic wounds and regenerative medicine is already visible in 2024–2026 literature [54,55,56,100,101]. Their eventual influence on post-procedural skin care may be substantial, but only after containment, immune compatibility, manufacturing and classification challenges are resolved.

AI could help connect discovery, cultivation, formulation and personalization. Useful applications include predicting how culture parameters affect metabolite quality, how a molecule behaves in a carrier, and which skin phenotype is most likely to respond. Such predictions require experimental validation before they can guide product development.

## 8. Conclusions

Microalgae and cyanobacteria are moving from niche cosmetic sources toward broader platforms for skin science. Their value comes from the combination of controllable photosynthetic production, unusual molecular diversity, and delivery modalities ranging from purified metabolites to extracellular vesicles and living oxygen-generating cells. EPS, MAAs, pigments, peptides/lipopeptides, and lipids already provide a broad molecular toolbox; nanoalgosomes and photosynthetic biomaterials extend that toolbox into biological delivery systems.

The central shift is from “algae as an ingredient” to “algae as a platform.” A useful product must connect a defined strain and manufacturing process to a characterized active, a skin-relevant mechanism, an appropriate delivery strategy, and a regulatory-compatible claim. Precise language is equally important: photoprotection is not automatically sunscreen activity, antimicrobial activity is not automatically a microbiome benefit, and antioxidant activity is not automatically anti-aging efficacy.

With this level of definition, algal dermocosmetics can contribute to preventive and supportive skin health while also informing innovations in wound repair, photoactivated therapy, and regenerative biomaterials. Progress will depend less on the number of new algal extracts reported than on how precisely their functions can be produced, delivered, measured, and translated.

The quantitative cultivation and separation examples also show that biomass yield, intracellular content, extraction recovery, and delivered potency are different measures. Growth-supporting and stress-induction strategies require different optimization, and modeled production costs remain conditional on scale and process boundaries. The next translational step is a linked mass balance, potency specification, and formulation-specific human assessment, rather than assuming that a higher laboratory yield or a more elaborate carrier guarantees a better skin product.

## Figures and Tables

**Table 1:** Representative microalgal and cyanobacterial bioactive classes for skin applications. Grades apply to the specified examples and endpoints, not automatically to an entire chemical class. A, controlled human intervention; B, uncontrolled human or human-tissue/reconstructed-skin evidence; C, animal or non-human organotypic evidence; D, cellular or biochemical evidence. Oral and topical routes are distinguished.

Bioactive Class	Representative Examples	Typical Source Organisms	Main Skin-Relevant Functions	References/ Evidence Level
Sulfated polysaccharides/EPS	secreted sulfated EPS; calcium spirulan; sacran; *Nostoc* EPS; arabino-rhamno-galactans; native and depolymerized EPS	*Porphyridium* spp.; *Arthrospira platensis*/maxima; *Aphanothece sacrum*; *Nostoc* spp.; *Chlorella vulgaris*; *Cylindrotheca closterium*; *Tetraselmis suecica*	hydration and film formation; barrier support; anti-inflammatory activity; bacterial anti-adhesion and antibiofilm effects; fibroblast migration; MMP-1 inhibition; collagen support; wound-supportive activity	[16,17,18,19,20,21,22,23]; A: selected controlled human endpoints; C–D: most mechanistic results
Mycosporine-like amino acids (MAAs)	palythine; porphyra-334; shinorine; mycosporine-glycine; mycosporine-2-glycine; 13-O-(β-galactosyl)-porphyra-334; MAA-rich fractions	*Sphaerospermopsis torques-reginae*; *Nostoc* spp.; *Nostoc sphaericum*; *Anabaena variabilis*; diatoms and other MAA-producing microalgae/cyanobacteria	UV absorption and photoprotection; limitation of UV-induced oxidative and DNA damage; anti-apoptotic effects; antioxidant activity; anti-glycation and anti-collagenase effects; wound-supportive activity	[24,25,26,27,28,29]; D: cellular/biochemical evidence
Carotenoids	astaxanthin; fucoxanthin; lutein; β-carotene; zeaxanthin	*Haematococcus pluvialis*; *Phaeodactylum tricornutum*; *Dunaliella salina*; *Chlorella zofingiensis*; *Tisochrysis lutea*	antioxidant and anti-inflammatory activity; NF-κB/MAPK/NLRP3 modulation; anti-photoaging; support of moisture and elasticity; pigmentation-related effects	[12,13,30,31,32,33,34]; C: topical animal models; A: selected oral trials; B: combined oral/topical exploratory data
Algal lipids/PUFAs	EPA- and DHA-rich lipids; glycolipids; phospholipids; polar lipid fractions	Nannochloropsis spp.; *Phaeodactylum tricornutum*; *Isochrysis*/*Tisochrysis* spp.; other oleaginous microalgae	barrier-lipid support; modulation of inflammatory tone; emollient activity; membrane-related effects; potential support of epidermal homeostasis	[35,36]; D/mechanistic rationale; no class-wide topical efficacy
Phycobiliproteins	C-phycocyanin; phycocyanin-rich fractions; other phycobiliproteins	*Arthrospira platensis*; *Synechococcus* spp.; other cyanobacteria	anti-inflammatory and antioxidant effects; soothing activity; topical skin deposition; wound-supportive effects	[37,38,39]; C: animal efficacy; D: cellular mechanisms
Bioactive peptides/protein hydrolysates	antimicrobial peptides; *Chlorella*-derived peptide; *Spirulina* protein hydrolysates; cyanobacterial peptides and lipopeptides	*Chlorella* spp.; *Arthrospira*/*Spirulina platensis*; *Synechococcus* spp.; diverse cyanobacteria and microalgae	antimicrobial and antibiofilm activity; modulation of MMP-1/procollagen pathways; fibroblast responses; angiogenesis, collagen deposition, and wound healing	[40,41,42,43,44,45]; C: nanoliposomal hydrolysate wound model; D: most molecular leads
Chlorophyll derivatives/photosensitizers	chlorophyll derivatives; pheophorbide *a* and related tetrapyrroles; pigment-rich photodynamic fractions	*Skeletonema marinoi* and other diatoms/microalgae	photoactivated antibacterial activity; antibiofilm effects; potential acne-directed phototherapy; localized ROS generation under controlled illumination	[46,47]; D: bacterial, biofilm and skin-cell assays
Scytonemin and distinctive secondary metabolites	scytonemin; related cyanobacterial UV-screening metabolites	*Nostoc* spp.; *Scytonema* spp.; other sheath-forming cyanobacteria	UVA screening; antioxidant effects; suppression of inflammatory signaling; inhibition of NF-κB-associated inflammatory mediators	[48,49,50,51,52]; C: scytonemin mouse inflammation; D: ScyM enzyme inhibition
Extracellular vesicles/nanoalgosomes	*Phaeodactylum* EVs; *Tetraselmis* nanoalgosomes; microalgal EV-loaded hydrogels	*Phaeodactylum tricornutum*; *Tetraselmis chuii*; Haematococcus-derived EV platforms	biomimetic cargo delivery; keratinocyte uptake; protection from oxidative and UVB damage; modulation of senescence and melanogenesis; anti-inflammatory and regenerative effects	[9,10,53]; C: Phaeodactylum EVs and EV hydrogel animal results; D: Tetraselmis skin-cell assays
Living photosynthetic cells/biomaterials	living microalgae in alginate or chitosan hydrogels; photosynthetic microneedles; photosynthetic pneumatic patches	*Chlamydomonas reinhardtii*; living microalgal and cyanobacterial systems	light-triggered local O_2_ generation; correction of wound hypoxia; sustained local delivery; wound healing and regenerative support	[11,54,55,56]; C: animal efficacy; B: healthy-volunteer tolerability of the alginate hydrogel only [11]

**Table 2:** Quantitative cultivation benchmarks illustrating growth-supporting and stress-induced production. Results are study-specific, not directly comparable industrial performance guarantees.

Target and System	Operating Strategy	Quantitative Result	Process Interpretation
C-phycocyanin and biomass; *Arthrospira platensis* F&M-C256/C260 [57]	6-L annular LED reactors; 700 µmol photons m^−2^ s^−1^; 35 °C; pH 9.2–9.4; 50% daily harvest in semi-continuous culture	Biomass: 0.94–0.95 g L^−1^ d^−1^ in semi-continuous operation versus 0.71–0.77 g L^−1^ d^−1^ in fed-batch. C-phycocyanin productivity: approximately 0.08 g L^−1^ d^−1^ in both regimes.	Maintain productive growth and target composition; artificial-light energy and optical path constrain scale-up.
Astaxanthin; *Haematococcus pluvialis* [58]	Semi-continuous green-stage supply followed by a separate induction stage; 40% renewal and 15-day induction in the reported optimum	Astaxanthin output: 9.6 mg per liter of green-stage semi-continuous culture per day	Denominator refers to green-stage culture volume; account for induction capacity before comparing whole-plant output.
Astaxanthin; *H. pluvialis* [59]	Independent optimization of green growth and red stress stages; irradiance adjusted to cell density	Two-stage indoor system: 4% dry-biomass astaxanthin and 11.5 mg L^−1^ d^−1^; 2.5–5-fold advantage over the compared one-stage systems	Benchmark supports phase-specific optimization under these conditions; it is not a universal superiority factor.

**Table 4:** Human-relevant and near-human evidence models used to support microalgal and cyanobacterial skin claims.

Model or Study Type	Representative Example	Measured or Proposed Endpoints	Interpretive Limit	References/Grade
Skin-cell cultures	Palythine; Chlorella peptide; Tetraselmis nanoalgosomes	UV injury; matrix markers; oxidative stress; melanogenesis	Human cell origin does not establish tissue penetration or clinical efficacy.	[10,25,44]; D
Animal skin or wound models	Liposomal astaxanthin; phycocyanin; EV hydrogel	Dermatitis; closure; collagen and inflammatory markers	Results are specific to the animal model and intervention.	[31,39,53]; C
Reconstructed human skin	Proposed validation step for defined algal formulations	Barrier function, irritation, photodamage under topical exposure	Model-development reviews do not themselves demonstrate algal efficacy.	[102,103]; B only when directly tested
Ex vivo human skin/skin-on-chip	Proposed exposure and response assessment	Retention, penetration, local pharmacodynamics	Relevance depends on tissue origin and validation; no algal efficacy inferred from the model alone.	[103,104,105,106]; grade conditional on actual model
Exploratory human tolerability	Living Chlamydomonas alginate dressing; 20 healthy volunteers	Local irritation and material compatibility	Does not demonstrate wound closure in patients.	[11]; B for tolerability
Controlled human intervention	Spiralin gel, *n* = 20; sacran serum; selected oral astaxanthin trials	Microbiome and clinical endpoints; corneocyte measures; skin biophysics	Small samples and route-specific outcomes; oral results cannot substantiate a topical product.	[12,13,23,99]; A by design, certainty assessed separately

**Table 5:** Representative delivery strategies and matched cutaneous targets for microalgal and cyanobacterial modalities.

Delivery Strategy	Material/Example	Target Compartment	Key Formulation Constraint	Evidence and Limits
Aqueous gel or serum	Spiralin; sacran; prospective MAA vehicles	Surface/upper epidermis	Preservation, compatibility, retained active concentration	[23,99]: A for specific tested products; [24,25,26,27,28,29]: D for MAA activity
Lipid carriers	Liposomal astaxanthin or phycocyanin	Surface and epidermal deposition	Oxidation, protein integrity, release; avoid equating retention with permeation	[31,37]: C for topical animal efficacy; no class-wide clinical carrier advantage
EV formulations	Phaeodactylum EVs; Tetraselmis nanoalgosomes	Skin-cell targets; intact-skin access must be measured	Particle integrity, cargo, isolation contaminants, preservative effects	[9]: C/D; [10]: D; no demonstrated clinical efficacy
EV-loaded hydrogel	Microalgal EV/tannic-acid system	Wound interface	Separate matrix, vesicle, and soluble-cargo effects	[53]: C; diabetic animal wound model
Living microneedles/pneumatic patches	Photosynthetic cells with co-therapeutics	Wound bed/postsurgical tissue	Viability, illumination, oxygen transport, containment	[54,55]: C; biomedical proof of concept
Living hydrogel	Chlamydomonas alginate dressing; composite hydrogels	Superficial interface/wound bed	Net oxygen supply under intended light and storage conditions	[11]: B for healthy-skin tolerability; [56]: C for wound efficacy

**Table 6:** Selected emerging microalgal and cyanobacterial skin-delivery innovations discussed in the review. Evidence levels follow Section 1.1 and indicate the highest directly relevant level for the stated skin/delivery application; where safety/tolerability and efficacy differ, both are specified.

Platform	Defined System/Model	Reported Advance	Main Remaining Limitation	References/Grade
Microalgal extract gel	Spiralin; paired lesions; 20 participants; 4 weeks	Shannon index +16.7% versus baseline at active sites; *p* < 0.01	*S. aureus* change not significantly superior to vehicle; exploratory clinical trends	[99]; A small controlled study
Astaxanthin nanocarrier	Liposomal astaxanthin; mouse dermatitis	Formulation-dependent anti-inflammatory activity	Human oral or combined-route studies do not validate this nanocarrier	[31]; C; oral context [12,13,34]
Phaeodactylum EVs	HaCaT cells and UV-exposed mouse skin	Protection against oxidative and UV-associated injury	Need standardized preparation and finished-formulation human testing	[9]; D in cells, C in mice
Tetraselmis nanoalgosomes	Human skin-cell cultures	UVB stress and senescence-associated changes; modulation of melanogenesis	Cellular data; intact skin delivery and clinical benefit remain unestablished	[10]; D
EV-loaded hydrogel	Microalgal EV/tannic-acid system; diabetic models	Wound and inflammatory/oxidative endpoints	Disentangle hydrogel and EV effects; establish scalable potency specification	[53]; C
Photosynthetic microneedles/patches	Postsurgical melanoma or deep-wound animal models	Local oxygenation combined with therapeutic delivery	Biomedical systems; no human efficacy inferred	[54,55]; C
Microalgae composite hydrogel	Photo-crosslinked Chlamydomonas/chitosan system	Preclinical wound repair	Co-therapy and material contributions require separate interpretation	[56]; C
Photosynthetic alginate dressing	20 healthy human volunteers	No irritation under the reported test protocol	Healthy-skin tolerability does not demonstrate healing of chronic wounds	[11]; B for tolerability only

## Data Availability

No new data were created or analyzed in this review. Data sharing is not applicable to this article.

## Abbreviations

The following abbreviations are used in this manuscript:

AI	artificial intelligence
EPS	exopolysaccharides
EVs	extracellular vesicles
LC-HRMS	liquid chromatography–high-resolution mass spectrometry
MAAs	mycosporine-like amino acids
MAPK	mitogen-activated protein kinase
MMP	matrix metalloproteinase
NF-κB	nuclear factor kappa B
NLRP3	NLR family pyrin domain containing 3
Nrf2	nuclear factor erythroid 2-related factor 2
OSMAC	One Strain Many Compounds
PUFAs	polyunsaturated fatty acids
ROS	reactive oxygen species
UVA	ultraviolet A
UVB	ultraviolet B
